# Influence of the argon admixture on the reactive oxide species formation inside an atmospheric pressure oxygen plasma jet

**DOI:** 10.1038/s41598-024-54111-y

**Published:** 2024-02-10

**Authors:** Ali Barkhordari, Saeed Karimian, Sajedeh Shahsavari, Dorota Krawczyk, Antonio Rodero

**Affiliations:** 1 Faculty of Physics, Shahid Bahonar University of Kerman, Kerman, Iran; 2 Department of Physics, Vali-e-Asr University of Rafsanjan, Rafsanjan, Iran; 3 Photonics Institute, Kerman Graduate University of Technology, Kerman, Iran; 4 Faculty of Civil Engineering and Environmental Sciences, Bialystok University of Technology, Bialystok, Poland; 5 Department of Physics, School of Engineering Sciences of Belmez, University of Cordoba, Cordoba, Spain

**Keywords:** Wire-to-multiwire dielectric barrier discharge, Fluid and global models, O_2_/Ar gas mixture, Applied physics, Plasma physics, Techniques and instrumentation

## Abstract

In this work, a new atmospheric pressure plasma generated in a wire-to-multiwire dielectric barrier discharge on pure oxygen is introduced. This special geometry of 13 wires (one central wire and 12 ones on the external tube) is feeding by a radio frequency (RF) power (13.56 MHz, 1 kW) and produces a stable discharge. The capacity of this device to produce oxygen reactive species and the influence of Ar gas mixture (1–3%) on this production are investigated. The main characteristics of this DBD plasma are measured using optical emission spectroscopy techniques. The rotational, vibrational, and excitation temperatures along with the electron density are determined from OH (A^2^Σ → X^2^Π) band and the Stark broadening of the hydrogen atomic line at 486.1 nm, respectively. The temporal evolution and spatial distribution of charged and reactive species in this plasma are also numerically studied by a Global scheme and a two-dimension fluid model based on drift–diffusion approximation. A kinetic dominated by electron collisions is obtained for this plasma. The generation and movement of electrons, positive and negative ions in the wire-to-multiwire configuration are analyzed and discussed according to changes the electric field and plasma frequency. It is shown that the density of both charged and reactive species increases by adding a small amount of argon to the oxygen plasma while the electron temperature reduces in this configuration. A high level of agreement is observed between the experimental and simulation results for the electron density and temperature in this DBD plasma.

## Introduction

It is well-known the high number of applications of the oxygen plasma, from the treatment and cleaning of surfaces to the biomedical field. The various components of plasma, such as electrons, ions, neutrals, molecular and atomic species, interact with the surface, altering its chemical and physical properties. Oxygen discharges have been applied for changing the properties of the surface of metals, plastics, glass or polymers^1,2^. Polymers are becoming increasingly popular due to their affordability and superior performance. Because of their low surface energy, which results in poor adhesion, their applications are limited. Functional groups formed by the oxygen plasma, are introduced into the surface, producing its modifications without disturbing the properties of the material^3^.

Surface modification can significantly enhance the adhesion of the polymer surface. Moreover, prior to growing an oxide mask layer or depositing an antireflection coating, it is essential to ensure that the surface is thoroughly cleaned in the case of microelectromechanical systems. The hydrophilic/hydrophobic properties of the surface or its poor adhesivity can be improved by the plasma treatment^4,5^. An atmospheric pressure oxygen plasma could be a convenient, in-line treatment process for cleaning, modifying, and activating the surface. So, it has been applied for photoresist stripping, removing polymer films, and oxidation or deposition of oxides film^6^.

Plasma treatment is a highly effective method for cleaning contaminants and modifying surfaces, making it ideal for a variety of applications. The oxygen plasma is also used for sterilization and cleaning of surfaces. The wet cleaning with chemicals such as detergents, solvents, acids, etc., raises problems of hazard waste control and the associated environmental and health risk. Dry-clean by plasma is a good alternative. The surface contaminant can be destroyed by thermal effect and/or oxidation by oxidative species produced in the discharge^2^. While the plasma method may have a high initial cost, its operating costs are lower than those of wet chemical cleaning techniques. For this, researchers have recently shifted their focus to plasma cleaning using atmospheric pressure plasmas due to the potential benefits it offers, such as eliminating the need for costly vacuum systems and components, enabling in-line processing, and allowing for scalability to larger areas.

In addition, use of oxygen plasmas is also interesting for biomedical applications, because of its capability to produce various biocidal agents including reactive species, UV radiation, and charged particles^7,8^. For all these applications determining the densities of oxidizing species and their dependences on various plasma parameters are of high importance. Additionally, it is essential to understand the densities of neutral species, particularly O radicals in oxygen plasmas, and how they are affected by various plasma or control parameters such as pressure, power, and dilution gas ratio^9^.

Low-pressure oxygen plasmas have been applied for these applications. Recently, special attention is paid to the atmospheric pressure plasmas due to their versatility, lower cost (no vacuum requirement), applicability to in-line processing, and the scalability to larger treatment area. Dielectric barrier discharges (DBD), Corona discharges, microwave plasma discharge, and atmospheric pressure torch are currently under investigation^10–13^. On the other hand, in recent years, the RF cold plasmas at atmospheric pressures have attracted much attention in various fields of science and technology^14–16^.

It is worth mentioning that the gas mixture plasma is potentially exciting for improving the plasma parameters, especially when the oxygen molecular gas, for example, is mixed with a rare gas such as argon atomic gas. Recently, the mixture of oxygen and argon gases has been used for increasing etching rate. It has been observed that when photoresist is ashed in an inductively coupled wave driven plasma source, the addition of argon to the oxygen discharge increases the plasma density and etch rate by a factor of two compared with a pure oxygen discharge. Despite this, the etching rate decreases significantly as the argon amount increases^17^.

In this work, a new RF driven atmospheric pressure plasma jet for working with O_2_ molecular gas is developed, that is based in a wire-to-multiwire configuration. This configuration allows to avoid the arc formation between electrodes during the ignition using a high gas flow (15 SLM). When the plasma is stabilized the gas flow can be reduced to 1 SLM. The oxygen and oxidative species produced inside plasma has been determined by Optical Emission Spectroscopy (OES). The dependence of the oxidative species formation on the Ar admixture was studied. To study the behavior of the effective species identified by OES technique along with the plasma processes, a theoretical study was performed, that allowed to determine the spatial and temporal distribution of species in the presented jet. Detailed fluid and kinetic descriptions of the plasma discharge in this configuration is established by PLASIMO software to characterize the main features of plasma. To this aim, the following steps are performed; (I) The temporal variations of the reactive species density will be calculated at three mixtures of O_2_/Ar gas by the global scheme, (II) Based on the drift–diffusion (DD) approximation, the spatial variations of the electron, positive and negative ions, electric field, electron temperature and frequency are obtained at 1 ms and three ratios of O_2_/Ar gas mixture. Both of them are well established methods to model the plasma discharge, the first one; based on the kinetic model, the second one; based on the fluid model. At the fluid model, the plasma is considered as a flow of charged particles. On the other hand, the global model is based on the kinetic of the particles including the neutral and charged species in the plasma. We can see the DD approximation as a spatio-temporal model that gives the spatial and temporal framework changes. The global model gives only the temporal variations in the volume of plasma discharge. The main difference of them is that the DD approximation could introduce the temporal changes in a specific point of plasma discharge while the global model represents an average value of any variables in the whole volume of the plasma discharge. Both results are useful at different situations^18^.

## Experimental setup

**Figure 1:**
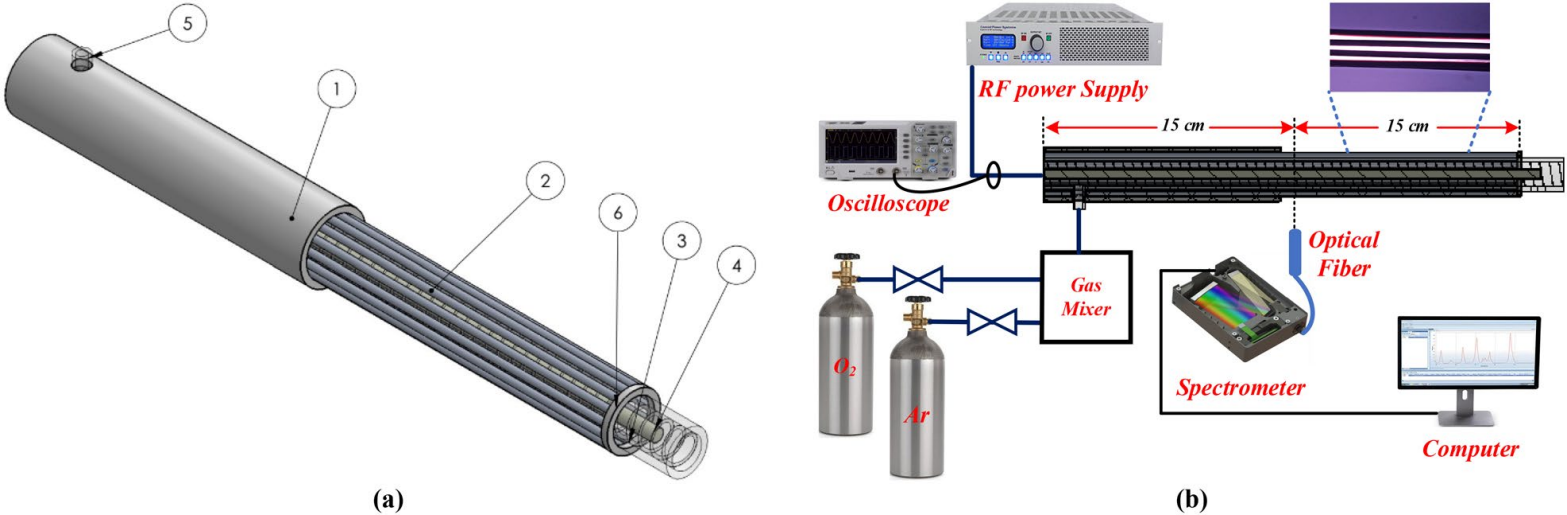
(**a**) 3D schematic of the designed plasma jet; (1) PTFE dielectric, (2) aluminum wires (ground electrode), (3) Quartz tube with spiral-shaped roughness in the inner wall, (4) Tungsten rod (power electrode), (5) Gas inlet, (6) PTFE dielectric. (**b**) Experimental setup.

As depicted in Fig. 1a for the designed RF plasma discharge, a central tungsten wire (inner) with 4 mm diameter has been placed at the center of a quartz tube with inner and outer diameters of 10 mm and 12 mm, respectively. Spiral-shaped roughness has been made in the inner wall of the quartz tube to move the gas as a vortex flow in the discharge medium. Due to this movement of gas flow, the temperature of the discharge medium is decreased by this roughness dielectric barrier. Moreover, 12 aluminum wires of 1 mm diameter have been cylindrically fixed around the tube as ground electrodes, with a separation of 30°. This device operates at atmospheric pressure with pure oxygen and small argon amounts mixed by oxygen. Thus, the plasma discharge is formed in a 1 mm gap between tungsten inner rode and aluminum outer wires that are covered by quartz dielectric. The length of generated RF plasma is about 20 mm along the inner electrode. This arrangement for plasma jet strongly prevents arcing between the inner and outer electrodes which is particularly unwanted for biomedical applications. Such a configuration for plasma jet generates a stable plasma discharge. The produced plasma transfers the reactive species to the processing region, which avoids disturbance to plasma stability.

In this work, a power supply with RF fixed-frequency (13.56 MHz) and constant output power (1 kW) has been applied. At first, both the output RF power and impedance are varied to reach the most stable plasma discharge for pure oxygen and all the its mixtures with argon. The impedance matching network works based on a transmission line and, a lumped L-type network has been used. It has to be noted that, the L-type matching network is lossless or, at least, the loss can be made extremely small with the proper component choices. However, the RF input power depends on the application and dimensions of the RF plasma jet.

At a DBD (AC or pulsed) plasma with high voltages, a breakdown occurs if the maximum voltage is reached. At atmospheric pressure, the streamer breakdown is contracted. Then, thin discharge channels appear, called filamentary. At RF discharges, penning ionization, stepwise ionization, and charge transfer collisions have a crucial effect on ionization dynamics that avoid the formation of this filamentary. So, a diffusive DBD or glow DBD is formed^19,20^.

In addition, two spectrometers are used for the spectroscopic characterization of this discharge. A HR4000 Ocean Optic spectrometer with optical resolution of width ~ 0.025 nm was applied for spectroscopic measurements. It stores a full spectrum at every millisecond with a wavelength range sensitivity of 200–1100 nm. Also, a spectrometer with higher resolution, Jobin Yvon, TRIAX550 with a resolution of 0.05 nm at 500 nm is used for the determination of plasma parameters, electron density and temperatures. It must be noted that, in order to calibrate these spectrometers, a HG-1 Mercury-Argon lamp was used as a light source that is capable of producing the spectral emission lines. Moreover, an optical fiber probe was placed at the middle of the plasma region (10 cm) which was perpendicular to the plasma discharge with a spatial distance of 5 mm.

It should be mentioned that the operational parameters of the external circuit are fixed. Using the gas mixer, the pure oxygen with 15 SLM (Standard Liters per Minute) gas flow rate, and O_2_/Ar gas mixture by 15 and 1–3 SLM gas flow rates enter the plasma jet during the ignition. When stable discharge is produced, a lower working flow of 1 SLM is used. It is worthy to mention that this flow rates are controlled using a velocity gas flow controller (Fig. 1b).

## Modeling

In this work, the effects of various mixtures of oxygen and argon on the spatial and temporal distributions of charged and reactive species, neutral particles, and the electron temperature in the wire-to-multiwire dielectric barrier discharge are theoretically studied when an RF power is used. The global scheme and 2D model are respectively based on the kinetic and fluid approaches which have been used to simulate O_2_/Ar gas mixture in the introduced electrode configuration.

### Global model

Let us consider a cylindrical chamber with a radius of R and a length of L into which the neutral species is entered by a steady flow via the gas inlet. It is assumed that the contents of the chamber are uniformly distributed at space, and the energy is uniformly deposited into the plasma. Moreover, electrons possess a Maxwellian-like energy distribution. Investigations of the electron energy distribution function (EEDF) reveal that it is largely consistent with the Maxwellian distribution in molecular plasmas^21^.

A continuity equation is used to describe the creation, gas phase and surface reactions, and losses of each species. So, a system of first order differential equations should be solved to evaluate the formation of reactive oxygen species originating from the oxygen molecules and the influence of adding argon atoms on the plasma processes in wire-to-multiwire dielectric barrier discharge considered in this work. The quasi-neutrality condition requires that the charged particle species be in balance:

1 $${n}_{{O}^{+}}+{n}_{{O}_{2}^{+}}+{n}_{{O}_{3}^{+}}+{n}_{{O}_{4}^{+}}+{n}_{{Ar}^{+}}+{n}_{{Ar}_{2}^{+}}={n}_{e}+{n}_{{O}^{-}}+{n}_{{O}_{2}^{-}}+{n}_{{O}_{3}^{-}}+{n}_{{O}_{4}^{-}}$$

where n refer to the number density of electrons and different positive and negative ions of oxygen and argon species considered in this study (see Table 1). In general, the time evolution of the number density of different species, *n*_*s*_, is expressed as^22–24^:

2 $$\frac{{dn}_{s}}{dt}=\sum_{i,j=1}^{{N}_{s}}({R}_{i,s}-{R}_{s,j})$$

where N_s_ is the number of reactions influencing on *s* specie, *R*_*i,s*_ and *R*_*s,j*_ refer to the reactions rate of the reactions that populate and depopulate this specie. Moreover, the reaction rate *R*_*i,j*_ between *i* and *j* species, depending on the density of each species, *n*_*i*_ and *n*_*j*_, with *k*_*i,j*_, the reaction constant, as follows^25,26^:

**Table 1:** The list of species considered in the model.

Neutrals	O, ${O}_{2}$, ${O}_{3}$,$Ar$
Pos. ions	${O}^{+}$, ${O}_{2}^{+}$, ${O}_{4}^{+}$, ${Ar}^{+}$,${Ar}_{2}^{+}$
Neg. ions	${O}_{2}^{-}$, ${O}_{3}^{-}$, ${O}_{4}^{-}$,${O}^{-}$
Elec. excited	${Ar}^{*}$, ${O}_{2}{[B}_{3}]$, ${O}_{2}[m]$, ${O}_{2}{[b}_{1}]$, ${O}_{2}{[a}_{1}]$, $O[1S]$,$O[1D]$
Vib. excited	${O}_{2}{v}_{1\dots 36}$

3 $${R}_{i,j}={k}_{i,j}{n}_{i}{n}_{j}$$

In this work, the reaction rate constants for electron collisions are calculated using the collision cross sections, *σ*_*i,j*_(*ε*), and EEDF, *f*(*ε*), by the following integral ^27^:

4 $${k}_{i,j}={\left(\frac{2q}{{m}_{e}}\right)}^{1/2}{\int }_{0}^{\infty }\varepsilon {\sigma }_{i,j}(\varepsilon )f(\varepsilon )d\varepsilon$$

with *ε* and *m*_*e*_ being the energy and electron mass. Otherwise, the reaction rate constants for heavy particles collisions are calculated by:

5 $$k=5.2\times {10}^{-47}exp(900/{T}_{g})$$

with T_g_ being the gas temperature. It is necessary to note the neutral species are diffused in the considered cylindrical chamber, and their diffusion coefficient is expressed as^28^:

6 $${D}_{n}=\frac{e{T}_{g}{\lambda }_{i}}{{v}_{n}{m}_{n}}$$

where T_g_ is the gas temperature, m_n_ is the mass of the neutral species, ${v}_{n}={(8e{T}_{g}/\pi {m}_{n})}^{1/2}$ is the mean speed of the neutral species, and ${\lambda }_{i}$ is the mean free path that is given by^29^;

7 $$\frac{1}{{\lambda }_{i}}=\sum_{j=1}^{{N}_{j}}{n}_{g,j}{\sigma }_{i,j}$$

with ${n}_{g,j}$ and ${\sigma }_{i,j}$ being the neutral species of jth ion and the scattering cross section of ion-neutral pairs for jth neutral species. In addition, the effective length of the diffusion of each neutral species in the cylindrical chamber with a length of R and a radius of R can be defined as follows^28^:

8 $${\Lambda }_{n}={[{\left(\frac{\pi }{L}\right)}^{2}+{(\frac{2.405}{R})}^{2}]}^{-1/2}$$

These quantities allow us to find the effective loss-rate coefficient required for examining the diffusional losses of the neutral species such as oxygen atoms, excited argon atoms, metastable oxygen atoms and molecules that reached the jet wall^29^;

9 $${k}_{n,\mathrm{ wall}}={[\frac{{\Lambda }_{n}^{2}}{{D}_{n}}+\frac{2V(2-{\gamma }_{n})}{A{v}_{n}{\gamma }_{n}}]}^{-1}$$

where V denote the volume of the cylindrical chamber, A is the surface area of the wall, and ${\gamma }_{n}$ being a coefficient for the neutral species stuck on the wall surface. On the other hand, the rate coefficient of the ion flux could be described as^29^:

10 $${k}_{+,\mathrm{ wall}}=2{u}_{B}\frac{{R}^{2}{h}_{L}+RL{h}_{R}}{{R}^{2}L}{s}^{-1}$$

where ${u}_{B}=\sqrt{e{T}_{e}/{m}_{i}}$ refers to the Bohm velocity, with m_i_ being the ion mass. Furthermore, the quantity of h_L_ and h_R_ denote the ratios of the positive ion density at the edge to center of the cylindrical chamber of plasma respectively given by^29^:

11 $${h}_{L}\approx 0.86(\frac{1+3\alpha /\gamma }{1+\alpha }){(3+\frac{L}{2{\lambda }_{i}})}^{-1/2}$$

12 $${h}_{R}\approx 0.80(\frac{1+3\alpha /\gamma }{1+\alpha }){(4+\frac{L}{2{\lambda }_{i}})}^{-1/2}$$

with $\alpha ={n}_{-}/{n}_{e}$ is the electronegativity with ${n}_{-}$ being the negative ion density, and $\gamma ={T}_{i}/{T}_{e}$. For the regime of $\gamma (R,L)\le {\lambda }_{i}\le (R,L)$, the above equations tend to Godyak's equations for an electropositive discharge because of $\alpha \to 0$^30^. In order to stabilize the modeling, the electron energy density equation should be solved together with the number density equation in simulation procedure which is described by^31^:

13 $$\frac{d{n}_{\epsilon }}{dt}=P-{Q}_{elas}-{Q}_{inelas}$$

where P is the input power density, *Q*_*elas*_ and *Q*_*inelas*_ are the energy loss and net energy loss due to the elastic and inelastic processes, respectively^32^. The power balance equation calculated by the ratio of the absorbed power P_abs_ and the power losses resulting from elastic and inelastic collisions as well as the charged particle flow to the walls, is defined as^33^:

14 $$\frac{{P}_{abs}}{V}=e{\mathcal{E}}_{c}^{\left(O\right)}{k}_{iz,O}{n}_{O}{n}_{e}+{k}_{w,{O}^{+}}e\left({\mathcal{E}}_{e}+{\mathcal{E}}_{i}\right){n}_{{O}^{+}}+e{\mathcal{E}}_{c}^{\left({O}_{2}\right)}{k}_{iz,{O}_{2}}{n}_{{O}_{2}}{n}_{e}+{k}_{w,{O}_{2}^{+}}e\left({\mathcal{E}}_{e}+{\mathcal{E}}_{i}\right){n}_{{O}_{2}^{+}}+e{\mathcal{E}}_{c}^{\left({O}_{4}\right)}{k}_{iz,{O}_{4}}{n}_{{O}_{4}}{n}_{e}+{k}_{w,{O}_{4}^{+}}e\left({\mathcal{E}}_{e}+{\mathcal{E}}_{i}\right){n}_{{O}_{4}^{+}}+e{\mathcal{E}}_{c}^{\left(Ar\right)}{k}_{iz,Ar}{n}_{Ar}{n}_{e}+{k}_{w,{Ar}^{+}}e\left({\mathcal{E}}_{e}+{\mathcal{E}}_{i}\right){n}_{{Ar}^{+}}+e{\mathcal{E}}_{c}^{\left({Ar}_{2}\right)}{k}_{iz,{Ar}_{2}}{n}_{{Ar}_{2}}{n}_{e}+{k}_{w,{Ar}_{2}^{+}}e\left({\mathcal{E}}_{e}+{\mathcal{E}}_{i}\right){n}_{{Ar}_{2}^{+}}$$

with ${\mathcal{E}}_{c}^{(l)}$ being the energy loss per electron–ion pair produced by the neutral which is given as ^33^:

15 $${\mathcal{E} }_{c}={\mathcal{E} }_{iz}+\sum_{i}{\mathcal{E} }_{ex,i}\frac{{k}_{ex,i}}{{k}_{iz}}+\frac{{k}_{el}}{{k}_{iz}}\frac{{3m}_{e}}{{m}_{i}}{T}_{e}$$

where T_e_ is the electron temperature, m_e_ is the electron mass, m_i_ is the ion mass. The coefficients of k_el_, k_iz_, and k_ex,i_ are the rate constant of elastic scattering, ionization, and ith excited state, respectively. Moreover, ${\mathcal{E}}_{ex,i}$ and ${\mathcal{E}}_{iz}$ refer to the energy of the ionization and the ith excitation processes, respectively^33^.

The system of these equations was solved Global Module of *PLASIMO.* It should be mentioned that this module works with *BOLSIG* + to use of the needed data of collisional electron reactions^32^. This model gives the time evolution of number density of *k* species that can be as a criterion of the lifetime of generated species in the plasma discharge.

In this model, 56 species presented in Table 1 have been considered which are resulted in 4000 reactions including electron impact, electron attachment, electron–ion recombination reactions, neutral–neutral reactions, ion-heavy particle reactions, vibrational energy transfer reactions.

### Two-dimension fluid model

#### Model domain

**Figure 2:**
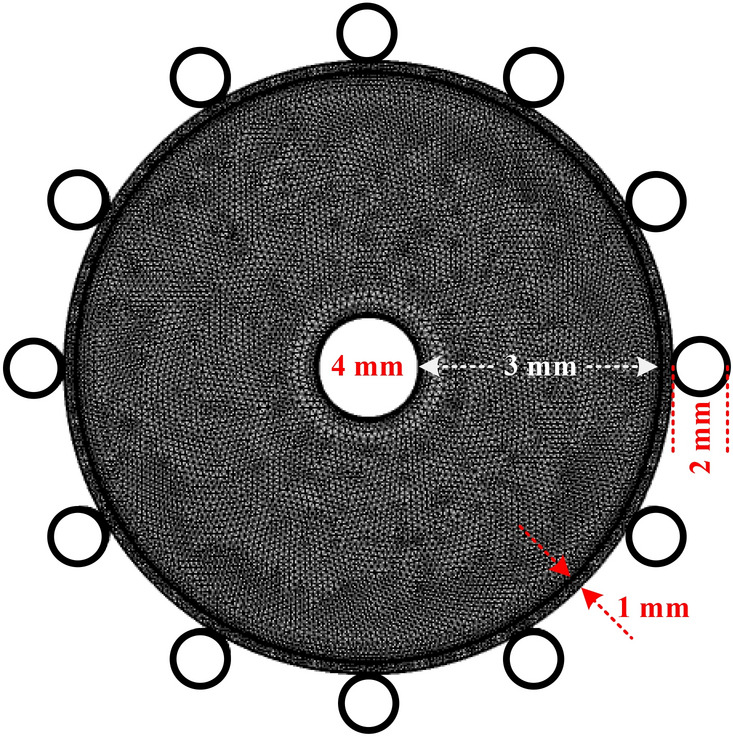
The simulation domain for wire-to-multiwire dielectric barrier discharge in the 2D model (the grid size is 2 nm).

Figure 2 shows a schematic of the domain which has been considered for simulation. As seen, a central tungsten wire with a diameter of 4 mm placed at the center of a 1 mm thick quartz tube was used as the power electrode. Moreover, 12 aluminum wires (2 mm diameter) were cylindrically fixed around the tube as ground electrodes. It is supposed that this structure operated at atmospheric pressure using both pure oxygen and a mixture of oxygen with small amounts of argon. Thus, the plasma formed in a gap of 3 mm between the tungsten inner rode and the aluminium outer wires covered by the quartz dielectric. Practically, this arrangement for discharge efficiently prevents arcing between the inner and outer electrodes, which is particularly unwanted for biomedical applications. Such a jet-type configuration generates a stable plasma. The produced plasma transfers the reactive species to the processing region, which avoids disturbance to plasma stability.

Due to cylindrical symmetry in the presented structure, the cylindrical coordinate is used to spatially simulate the 2D fluid model. To this aim, a symmetry along the z-direction is considered by assuming being in the middle of discharge (a large distance from two end of wires). Then the spatial description of the problem is only defined in radial and azimuthal directions. Because of using the fluid model to spatially simulate, only three charged species (electrons, positive and negative ions) are taken into account. In following, the fluid and the global models are briefly described.

#### Model equations

The spatial and temporal macroscopic description of the gas discharge inside the wire-to-multiwire configuration is via solving the fluid continuity equations for different species coupled with Poisson’s equation. These equations based on drift–diffusion approximation are solved using the finite difference (FD) method. The flux for each species in the drift–diffusion approximation is based on the momentum conservation of each species which is defined as^29^:

16 $${{\varvec{\Gamma}}}_{i}=\pm {\mu }_{i}{n}_{i}{\varvec{E}}-{D}_{i}{\nabla n}_{i}$$

where *μ*_*i*_ is the electrical mobility, *D*_*i*_ is spatial diffusivity, ***E*** is the electric field, and *n*_*i*_ is the number density of species i. The plus or minus sign in this relation accounts for the sign of the charged particles^34^. The continuity equation for all the formed species in the plasma discharge is expressed as^25^:

17 $$\frac{\partial {n}_{i}}{\partial t}+\nabla \cdot {{\varvec{\Gamma}}}_{i}=\sum_{m}{R}_{i,m}$$

where *n*_*i*_ is the number density, **Γ**_i_ expresses the flux for species *i* and *R*_*i,m*_ is the reaction rate between species *i* and species *m*. In this model, the kinetic scheme is the same that the used for global model (see table in supplementary material).

Moreover, the rate of change of the electron energy density is described by^29^:

18 $$\frac{{\partial n}_{\varepsilon }}{\partial t}+\nabla \cdot [-{\mu }_{\varepsilon }{n}_{\varepsilon }{\varvec{E}}-{D}_{\varepsilon }{\nabla n}_{\varepsilon }]+e{\varvec{E}}\cdot {{\varvec{\Gamma}}}_{e}={R}_{\varepsilon }$$

where *n*_*ε*_ is the electron energy density, $e{\varvec{E}}\cdot {{\varvec{\Gamma}}}_{e}$ is the ohmic or joule heating for electrons, *R*_*ε*_ is the energy loss or gain due to inelastic collisions which is obtained by summing the collisional energy loss or gain over all reactions^25^:

19 $${R}_{\varepsilon }=\sum_{j=1}^{P}{x}_{j}{k}_{j}{N}_{n}{n}_{e}{\Delta \varepsilon }_{j}$$

where *x*_*j*_ is the mole fraction of the target species for reaction *j*, *k*_*j*_ is the rate coefficient for reaction *j*, *N*_*n*_ is the total neutral number density and Δ*ε*_*j*_ is the energy loss from reaction *j*.

For non-electron species, the following equation is solved for the mass fraction of each species^35^:

20 $$\rho \frac{{\partial \xi }_{k}}{\partial t}+\rho \left({\varvec{u}}\cdot \nabla \right){\xi }_{k}=\rho (\nabla \cdot {{\varvec{V}}}_{k}){\xi }_{k}+{R}_{k}$$

where *ρ* denotes the density of the mixture, *ξ*_*k*_ is the mass fraction of the *k*th species, ***u*** is the mass averaged fluid velocity vector, ***V***_*k*_ and *R*_*k*_ are the multicomponent diffusion velocity and the rate expression for species *k*, respectively^35^. In a diffusion model, the multicomponent diffusion velocity, ***V***_*k*_, can be calculated by the Fick’s law^35^:

21 $${{\varvec{V}}}_{k}={D}_{k,f}\frac{\nabla {\xi }_{k}}{{\xi }_{k}}+{D}_{k,f}\frac{\nabla M}{M}+{D}_{k}^{T}\frac{\nabla T}{T}-{z}_{k}{\mu }_{k,m}{\varvec{E}}$$

where ${D}_{k,f}$ and ${D}_{k}^{T}$ are the diffusion coefficient and thermal diffusion coefficient for each species, *M* is the mean molar mass of the mixture, T is the gas temperature, and ${z}_{k}$ and ${\mu }_{k,m}$ represent the charge and mobility of species *k*. This mobility is given by the Einstein’s relation^35^:

22 $${\mu }_{k,m}=\frac{{z}_{k}{D}_{k,f}}{{k}_{B}T}$$

The energy balance for heavy particles is calculated by Fourier`s equation that allows to obtain gas temperature *T*:

23 $$\rho {C}_{p}\frac{\partial T}{\partial t}=\nabla \cdot \left(k\nabla T\right)+R$$

where *k* and ${C}_{p}$ are the thermal conductivity and specific heat capacity of oxygen gas, respectively, and R is the heat sources, originating from the Joule effect and collisions among electron and heavy particles^35^. In the model, the axial energy transport is considered to be neglected because low working gas flow is used. It should be highlighted that in a discharge of molecular gas, the fast-heating effect by quenching of excited molecules with neutral or atomic species could be vital in plasma heating. It becomes more crucial in air discharge by quenching N_2_ excited states and oxygen molecules^36–38^. This effect is neglected in the studied plasma discharge because the amount of nitrogen is too weak. Although the quenching of O(^1^D) oxygen occurs in pure oxygen plasma, this effect is relevant for high energy density, as in the case of nanosecond discharge^37^. The RF discharge is far from these conditions. So, we can neglect the fast heating in our model.

To initiate dielectric barrier discharge in the wire-to-multiwire structure, electric potential should be applied between the electrodes, thus the Poisson’s equation must also be considered in the model^25^:

24 $$\nabla .(\varepsilon \nabla \varphi )=-e(\sum_{k=1}^{k}{Z}_{k}{n}_{k}-{n}_{e})$$

where *φ* is the electric potential, ${\varepsilon (=\varepsilon }_{0}{\varepsilon }_{r})$ is the permittivity (${\varepsilon }_{0}$ for vacuum and ${\varepsilon }_{r}$ for the dielectric), *n*_*k*_ and *Z*_*k*_*e* denote the density of charged species and their charge, respectively^39^.

#### Boundary conditions

To obtain a unique solution for the coupled equations system with the geometry presented in Fig. 1, necessary boundary conditions must be imposed. The applied boundary conditions for the wire-to-multiwire dielectric discharge are similar to those that can be found in the existing literature^40^. The following boundary condition is used to account for the particles flux in wall:

25 $${\varvec{n}}\cdot {{\varvec{\Gamma}}}_{{\varvec{i}}}=\left({a}_{i }sgn\left({q}_{i}\right){\mu }_{i}({\varvec{n}}\cdot {\varvec{E}})+\frac{1}{4}{v}_{th(i)}\right){n}_{i}$$

where ***n*** is the normal vector pointing toward the tube wall and, *v*_*th*(*i*)_ is the thermal velocity of particles^41^:

26 $${v}_{th(i)}= \sqrt{\frac{8{k}_{B}}{\pi }\frac{{T}_{i}}{{m}_{i}}}$$

and the number *a*_*i*_ is defined by:

27 $${a}_{i}=\left\{\begin{array}{c}1 \,sgn\left({q}_{i}\right){\mu }_{i}n\cdot E\ge 0\\ 0 \,sgn\left({q}_{i}\right){\mu }_{i}n\cdot E\le 0\end{array}\right.$$

For electrons, as a special case, the particles flux due to secondary electron emission (SEE) is added to the system and is defined as follows^40^:

28 $${\varvec{n}}\cdot {{\varvec{\Gamma}}}_{e}=\left(-{a}_{e}{\mu }_{e}({\varvec{n}}\cdot {\varvec{E}})+\frac{1}{4}{v}_{th,e}\right){n}_{e}-\sum_{p}{\gamma }_{p}{({\varvec{n}}\cdot \boldsymbol{ }{\varvec{\Gamma}}}_{p})$$

where *γ*_*p*_ are the SEE coefficients, which defines the average number of electrons emitted per impact of ions *p* on the tube wall. Similarly, boundary condition for electron energy is^41^:

29 $${\varvec{n}}\cdot {{\varvec{\Gamma}}}_{\varepsilon }=-\frac{5}{6}{v}_{th,e}{n}_{\varepsilon }-\sum_{p}{\gamma }_{p}{\varepsilon }_{p}({\varvec{n}}\cdot {{\varvec{\Gamma}}}_{p})$$

Here, the second term is the SEE energy flux, being *ε*_*p*_ the mean energy of the secondary electrons. The plasma discharge is driven by an RF potential applied to the wire centered at the axis of quartz tube as

30 $$\varphi ={\varphi }_{RF}sin\left(2\pi {f}_{RF}t\right)$$

and other electrodes located on the tube are grounded. It should be mentioned that ${\varphi }_{RF}$ is the amplitude of RF voltage used to initiate the plasma discharge. The electric displacement vector, ${\varvec{D}}=\varepsilon {\varvec{E}}$, on the dielectric tube is changed as follows:

31 $${\varvec{n}}\cdot ({{\varvec{D}}}_{2}-{{\varvec{D}}}_{1})={\rho }_{s}$$

and the surface charge density, *ρ*_*s*_, on the dielectric surface is given by:

32 $$\frac{\partial {\rho }_{s}}{\partial t}={\varvec{n}}\cdot {{\varvec{j}}}_{{\varvec{i}}}+{\varvec{n}}\cdot {{\varvec{j}}}_{{\varvec{e}}}$$

where ***j***_***e***_ and ***j***_***i***_ are the total electron and ion current densities on the dielectric layer^40^. Unless otherwise mentioned, the simulations have been performed with the chosen parameters presented in Table 2. It has been tried to select the parameters close to the real experimental values of atmospheric plasma discharge. The reactions used in the 2D fluid model have been presented in Appendix (see Table S1). It must be noted that the drift–diffusion module of *PLASIMO* has been applied to do the 2D simulation.

**Table 2:** Parameters used in the simulation.

Symbol	Value
Initial gas temperatures (eV)	0.026
Pressure (atm)	1
RF voltage amplitude (V)	500
RF frequency (MHz)	13.56
Initial electron density (cm^−3^)	10^11^
Total time (ms)	1
Time-steps (ps)	1

## Results and discussion

### Spectroscopic results

**Figure 3:**
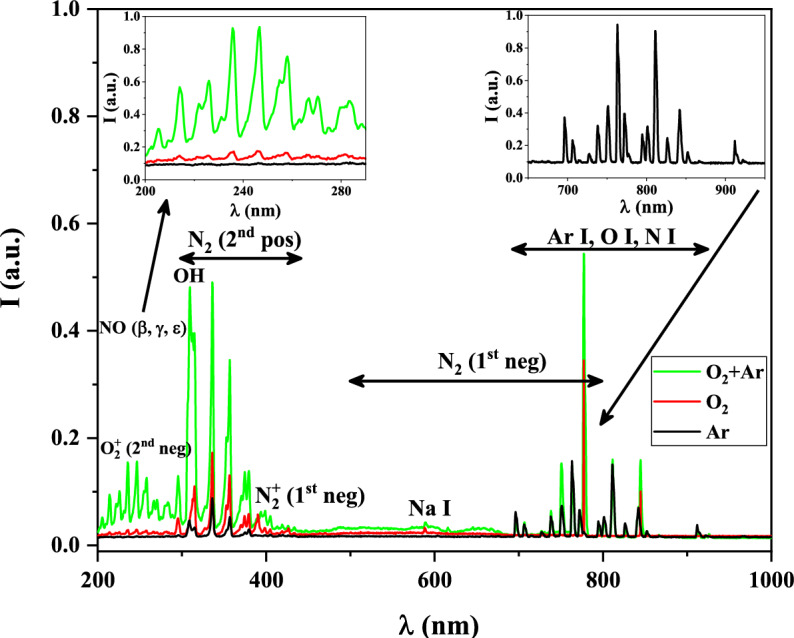
Emission spectrum of wire-to-multi-wire plasma jet with 3% Ar in O_2_/Ar gas mixture at the middle of plasma (15 cm).

**Figure 4:**
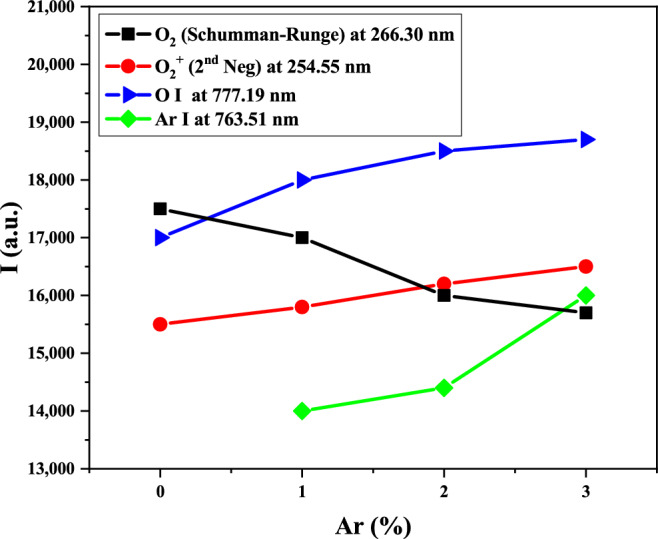
Variation of the relative intensity of species in the discharge with the Ar concretion in the O_2_/Ar admixture.

By analyzing the spectra recorded at various percentages of Ar in the O_2_/Ar gas mixture, different species generated in the wire-to-multiwire DBD plasma at atmospheric pressure are determined. Figure 3 shows both typical emission spectrum of pure O_2_ and O_2_ + 3%Ar plasmas and 1 kW RF power. It must be noted that the tube of this plasma reactor was opened at its end, which allowed some air went into it and reached the plasma. Thus, some excited nitrogen-containing species were detected in the spectra including N_2_ and NO molecules, N_2_^+^ ions, and nitrogen atoms. This nitrogen entrance could produce heating of the discharge, especially in the outside flame, and influence in the application of this type of plasma. We are considering neglected this effect because we study the oxidative specie formations inside the reactor. Water molecules are usually present in ambient air and in the gas as impurity, which explains the presence of OH radicals and hydrogen atoms in the spectra. On the other hand, some excited tungsten and sodium atoms were also detected, revealing that some tube and inner electrode etching took place. The gas temperature in this reactor is near to the ambient temperature (300 K). Then this etching could only be explained by the collisions of accelerated electron and ions with the quartz tube. Details of the different excited species produced in the plasmas are gathered in Table 3. Moreover, variations of the intensity of O_2_ (Schumman-Runge), O_2_ (2nd Neg), O I, and Ar I species at the wavelength of 266.33 nm, 254.55 nm, 777.19 nm, and 763.51 nm are represented in Fig. 4. The (H_α_) and (H_β_) visible spectral lines in the Balmer series of hydrogen atoms have been detected at the wavelengths of 656.1 nm and 486.01 nm, respectively^42^. These lines can be used to determine the electron density in the plasma discharge by calculating Stark broadening which is following discussed in details.

**Table 3:** Detected peaks for the RF plasma jet with O_2_/Ar gas mixture^42,43^.

Species	$\lambda$ (nm)	Transition symbol
NO (β system)	202.11, 210.36	${{\text{A}}}^{2}\Sigma \to {{\text{X}}}^{2}\Pi$
NO (γ system)	203.07, 205.28, 214.91, 222.24, 226.28, 230.95, 236.33, 247.11, 255.00, 258.75, 263.07, 267.14, 271.32, 275.52, 280.08, 288.22	${{\text{A}}}^{2}{\Sigma }^{+}\to {{\text{X}}}^{2}\Pi$
NO (ε system)	209.98	${{\text{D}}}^{2}{\Sigma }^{+}\to {{\text{X}}}^{2}\Pi$
OH (3064 °A system)	308.90	${{\text{A}}}^{2}{\Sigma }^{+}\to {{\text{X}}}^{2}\Pi$
${N}_{2}$ (2nd pos)	295.32, 296.20, 287.68, 315.93, 337.13, 357.69, 371.10, 375.54, 380.49, 399.84, 405.94, 409.48, 414.18, 426.97, 434.36, 457.43, 472.35, 481.47, 487.64, 491.68	${{\text{C}}}^{3}\Pi \to {{\text{B}}}^{3}\Pi$
${N}_{2}$ (1st neg)	500–800	${B}^{3}\Pi \to {{\text{A}}}^{3}\Sigma$
${N}_{2}^{+}$ (1st neg)	385.79, 388.43, 391.44	${B}^{2}{\Sigma }_{u}^{+}\to {X}^{2}{\Sigma }_{g}^{+}$
${O}_{2}$ (Schumman-Runge)	266.30	${B}^{3}{\Sigma }_{u}^{-}\to {X}^{2}{\Sigma }_{g}^{-}$
${{\text{O}}}_{2}^{+}$ (2nd neg)	205.97, 210.37, 222.43, 225.28, 230.72, 235.43, 246.58, 254.55, 258.10, 266.65, 270.53, 282.37, 283.97, 289.03	^2^Π → ^2^Π
O I	615. 60, 615.67, 615.82 777.19, 777.42, 777.54, 844.63, 844.64, 844.68	$4d\to 3p$ $4p\to 3s$
Na I	351.11, 418.51 589.00, 589.59	$3s3d\to 3s3p$ $3p\to 3s$
N I	856.78	$3p\to 3s$
Ar I	696.54, 706.72, 714.70, 727.29, 738.40, 750.39, 751.47 763.51, 772.38, 772.42, 794.82, 800.62, 801.48, 810.37, 811.53, 826.45, 840.82, 842.46, 852.14, 866.79, 912.30, 922.45	$4p\to 4s$
${H}_{\alpha }$	656.2	$3d\to 2p$
${H}_{\beta }$	486.1	$4d\to 2p$

#### Rotational, vibrational and excitation temperatures

The electron and ions are responsible of energy transferring from the electric field. Electrons get the energy from this field and transferred to neutrals and ions by elastic and inelastic collisions. Inelastic collisions with the molecules can produce the excitations of the ro-vibrational states, and also the excitation of electronic levels of these molecules if the electrons have enough energy. The rotational, vibrational, and excitation (or electronic) temperatures, *T*_*rot*_, *T*_*vib*_, and *T*_*exc*_, give the distribution of rotational, vibrational and electronic states of the molecules, considering a Boltzmann distribution. These temperatures can be obtained using the OES technique which is characterized from the energy levels of excited states. To examine the rotation, vibration, and excitation temperatures in the wire-to-multiwire DBD plasma, the OH (${{\text{A}}}^{2}\Sigma \to {{\text{X}}}^{2}\Pi$) band is chosen in the emitted spectra of the plasma discharge at atmospheric pressure. Hence, the simulated spectra are obtained and, to estimate the rotation, vibration, and excitation temperatures, they are fitted with the experimental emission spectra using the SPECAIR software^43,44^. In this simulation work, all the affected parameters are considered as the instrumental resolution and line shape with the collisional broadening.

**Figure 5:**
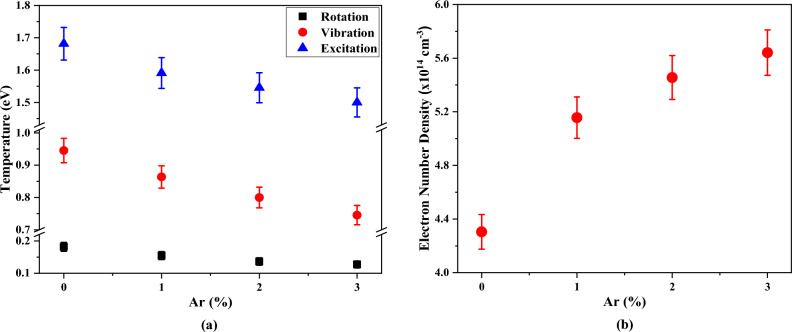
Variations of (**a**) electron number density (with 4% error) and (**b**) rotation, vibration, and excitation temperatures as a function of Ar fraction in O_2_/Ar mixture at position 5 mm (with 4% error).

Using the ro-vibrational spectrum of ·OH radicals at the wavelength of 309.1 nm (${{\text{A}}}^{2}\Sigma \to {{\text{X}}}^{2}\Pi$ transition), the rotational, vibrational, and excitation temperatures can be obtained^45^. Variations of all temperatures in terms of Ar contributions in the O_2_/Ar gas mixture are illustrated in Fig. 5a. As can be seen, all temperatures are intensively reduced by adding Ar to the O_2_ plasma and these reductions are continued by increasing Ar amounts in the O_2_/Ar gas mixture. The values of *T*_*rot*_ and *T*_*vib*_ are lower than the *T*_*exc*_. In equilibrium conditions the *T*_*rot*_ is assumed close to the gas temperature *T*_*g*_ and the *T*_*exc*_ close to the electron temperature, *T*_*e*_,. In non-equilibrium conditions, the following relation is followed:

33 $${T}_{g} \le {T}_{rot}<{T}_{vib}<{T}_{exc}\le {T}_{e}$$

#### Electron density

The electron density of this plasma was determined from the OES technique based on the Stark broadening of hydrogen Balmer series H_β_ line measurement. For this determination, they considered the different interaction mechanisms causing the broadening of this line, whose profile can be considered Voigt-type, resulting from the convolution of a Gaussian and a Lorentzian profile^46^,

34 $${\Delta \lambda }_{D}\left(nm\right)=7.2\times {10}^{-7}\sqrt{\frac{{T}_{g}}{M}}\lambda$$

where M is the atomic weight of hydrogen in atomic mass unit, T_g_ the gas temperature in K, and λ = 486.1 nm. The instrumental broadening, Δλ_I_, due to the spectroscopy detection system was considered equal to 0.05 nm. Both determine the broadening of the Gaussian profile of the line whose broadening is given by^47^:

35 $${\Delta \lambda }_{G}=\sqrt{{\Delta \lambda }_{D}^{2}+{\Delta \lambda }_{I}^{2}}$$

On the other hand, the collisional mechanisms leading to the Lorentzian profile in this case are:

(i) the Van der Waals broadening, Δλ_van der Waals_, given by the following expression calculated as in ref.^41^, considering for this case H_2_O molecules as perturbers of H atoms emitters^42,46^:

36 $${\Delta \lambda }_{van der Waals}=8.18\times {10}^{-26} {\lambda }^{2}{(\alpha \langle {R}^{2}\rangle )}^{0.4}{\left(\frac{{T}_{g}}{\mu }\right)}^{0.3}\frac{P}{{k}_{B}{T}_{g}}$$

where µ is the reduced mass of the colliding particles (µ = 0.94737 for H/H_2_O collision), λ is the wavelength of the H_*β*_ line (486.1 nm), α is the molecular polarizability of the H_2_O disturbing particles (α = 1.43 × 10^–24^ cm^3^), 〈R^2^〉 is the difference of the square radius of the upper and lower levels of H_*β*_ transition, T_*g*_ is the gas temperature in K, and P is the pressure (1 atm for atmospheric pressure).

(ii) the Stark broadening, Δλ_*Stark*_, which is related to the electron density^48^:

37 $${\Delta \lambda }_{stark}(nm)=2\times {10}^{-11}({n}_{e}^{2/3})$$

The resonance broadening, due to the dipole–dipole interactions of the emitters with the ground-state atoms of the same element, can be neglected in this particular case where just small amounts of hydrogen are present in the plasma. Thus, the Stark and van der Waals broadenings determine a Lorentzian profile whose broadening is expressed as^47^:

38 $${\Delta \lambda }_{Lorentz}(nm)={\Delta \lambda }_{Stark}+{\Delta \lambda }_{van \,der \,Waals}$$

Finally, every emission line profile is considered as Voigt shaped (resulting from the convolution of the Gaussian and Lorentzian functions) with a broadening given by^48^:

39 $${\Delta \lambda }_{V}=\frac{{\Delta \lambda }_{L}}{2}+\sqrt{{\left(\frac{{\Delta \lambda }_{L}}{2}\right)}^{2}+{\Delta \lambda }_{G}^{2}}$$

Therefore, a numerical fitting of the experimental spectrum of the line to a Voigt function allows us to discriminate Lorentzian and Gaussian contributions. Thus, once measured Δλ_L_ and determined Δλ_VdW_ contribution from (36) (using the value of T_g_ previously measured), the Stark broadening of H_β_, and consequently the electron density, can be obtained (Eq. 37). In this work, the Microcal Origin software was used to perform the fitting of the line to a Voigt profile. As shown in Fig. 5b, the electron number density increases at the higher argon contributions. In O_2_/Ar gas mixture, part of the energy applied to the discharge is used for the excitation and decomposition process of O_2_ molecules. Thus, ionization and electron production are reduced when the concentration of these molecules is increased.

### Formation of species in the plasma

**Figure 6:**
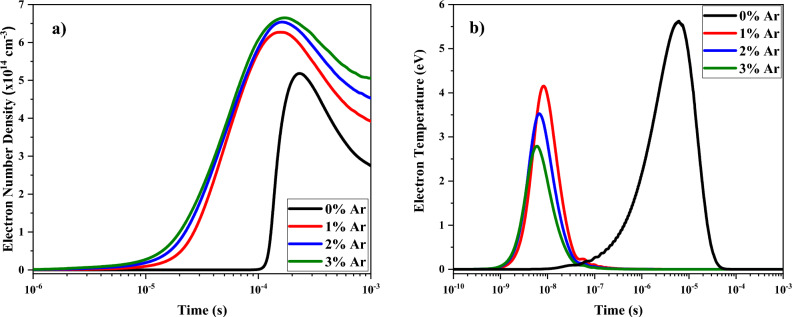
Temporal variations of electron (**a**) density and (**b**) temperature at different percentage of argon mixed by oxygen.

The temporal evolution and formation of species in the plasma discharge was studied by the Global Model. Figure 6a shows the results of the temporal variations of the electron density and temperature for pure oxygen plasma and for different Ar admixture. As can be observed, the electrons start to be produced in earlier times when the Ar is added. It is due to the argon needs less electrical energy for its ionization. When the electrons are produced, they accelerate by the electric field and produce new ionizations. By the same motive, at higher contents of argon in the O_2_/Ar gas mixture, the number density of electrons produced in the plasma discharge is increased. In addition, the electron number density evolution presents a maximum value in 0.1 ms and then drops to reach a stable value in all considered mixtures. The increase of electron number density in the plasma discharge results in more dissociation and creation of different oxygen species due to collisional ionization processes. This behavior and density values agree with the experimental measures of electron density obtained in Fig. 5b.

On the contrary, the electrons temperature is significantly reduced at higher fractions of argon in the O_2_/Ar gas mixture as shown in Fig. 6b. It is due to the energy supplied by the electric field is distributed between the higher number of electrons. Like the electron density, the temporal evolution of electron temperature has a maximum in earlier times. But the position of this maximum is in shorter times than the electron density case. When the electron number starts to increase, the average shared energy is lower and consequently the electron temperature decreases. Also, the temperature starts to rise in earlier times as the Ar concentration is increased. The behavior of electron temperature agrees also with experimental measurements. The temperatures decrease when Ar is added to the discharge. However, values of this temperature are higher than those obtained ones by spectroscopic measurements (see Fig. 5a). This difference is due to the separation of the excitation states from the electronic states of molecules by electrons at non-equilibrium conditions, shown in Eq. (33).

**Figure 7:**
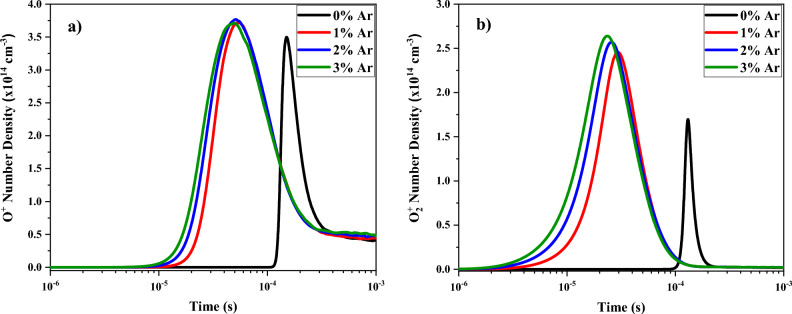
Temporal variations of density profile for (**a**) O^+^ and (**b**) ${O}_{2}^{+}$ at different percentage of argon mixed by oxygen.

Figure 7 shows the temporal changes of the positive ions number density, i.e., O^+^ and ${O}_{2}^{+}$, at various contributions of argon added to oxygen plasma. At higher percentages of argon in the O_2_/Ar gas mixture, the number density of ${O}_{2}^{+}$ is decreased while the most amount of the O^+^ number density is resulted in 2% of Ar in the gas mixture. Besides, the number density of O^+^ species has been grown up at all gas mixtures in respect to the pure oxygen. It must be noted that, in addition to increasing of the O^+^ and ${O}_{2}^{+}$ production, the permanence time of these species is significantly increased by adding argon gas to the oxygen plasma. As can be seen, the durability is also raised at more contents of argon in the gas mixtures.

This temporal behavior is consequence that the main sources of the O^+^ and ${O}_{2}^{+}$ positive ions production are the collisions of oxygen molecules with electrons (Reactions 11 and 13 in Table S1):

40 $$\begin{aligned} & e+{O}_{2}\to e+e+O+{O}^{+} \\ & e+{O}_{2}\to e+e+{O}_{2}^{+} \end{aligned}$$

So, this temporal distribution of formed ions has a direct relation with the number of electrons in the discharge. Nevertheless, the position of the maximum is a bit lower than the electron number density. This is due to the recombination and dissociative recombination processes by electron collisions start to be also important when the electron density is enough high (Reactions 20 and 48 in Table S1) and the positive ions density is consequently reduced:

41 $$\begin{aligned} & e+{O}_{2}^{+}\to O+O \\ & e+{O}^{+}+{O}_{2}\to O+{O}_{2} \end{aligned}$$

**Figure 8:**
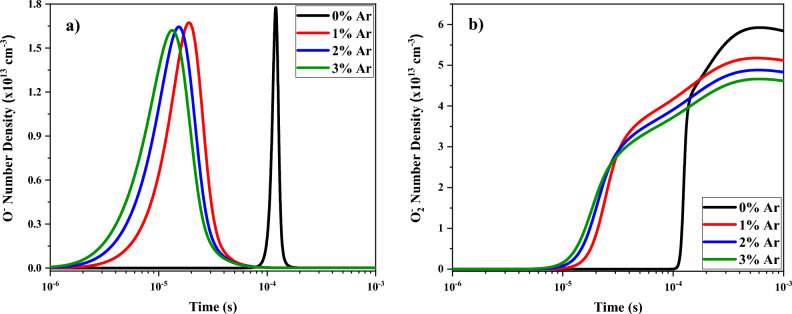
Temporal variations of density profile for (**a**) ${O}^{-}$ and (**b**) ${O}_{2}^{-}$ at different percentage of oxygen mixed by argon.

The temporal changes of the number density of the negative ions of oxygen atom and molecule, i.e., ${O}^{-}$ and ${O}_{2}^{-}$ at different argon contents added to oxygen plasma are shown in Fig. 8. When argon is added to the pure oxygen, a broader peak is again obtained and the ${O}^{-}$ and ${O}_{2}^{-}$ species are generated earlier. However, the maximum number density of the ${O}^{-}$ and ${O}_{2}^{-}$ species is lightly lower at the O_2_/Ar gas mixtures than the pure oxygen and the maximum position is earlier than the positive ions (see Fig. 7).

Again, the negative ions ${O}^{-}$ are mainly produced by electrons collision with oxygen molecules (Reactions 8 and 9 in Table S1):

42 $$\begin{aligned} & e+{O}_{2}\to e+{O}^{-}+{O}^{+} \\ & e+{O}_{2}\to {O}^{-}+O \end{aligned}$$

Then these ions start to be generated as the electron density is increased. But their reactions with:

Electrons:

43 $$e+{O}^{-}\to e+e+O$$

Positive ions:

44 $$\begin{aligned} & {O}^{-}+{O}_{2}^{+}\to {O}_{2}+O \\ & {O}^{-}+{O}_{2}^{+}\to 3O \\ & {O}^{-}+{O}^{+}\to 2O \end{aligned}$$

And neutral species:

45 $$\begin{aligned} & {O}^{-}+O\to {O}_{2}+e \\ & {O}^{-}+O\to {O}_{2}+e \\ & {O}^{-}+{O}_{2}\to {O}_{3}+e \end{aligned}$$

can produce their losses. Consequently, the density of negative ions decreases when the amount of all species is enough high.

Also, Fig. 8 shows that the shape of the temporal evolution of positive ions is more influenced by the Ar concentration than previous cases. It is due to the negative ions can be also destroyed by collisions with ions Ar^+^:

46 $${Ar}^{+}+{O}^{-}\to Ar+O$$

The ${O}_{2}^{-}$ species are generated by the collision between the negative ion of O atoms and excited species of O_2_ molecules in addition to the electron collisions with the neutral species of O_2_ molecules as:

47 $$\begin{aligned} & {O}^{-}+{O}_{2}^{*}\to {O}_{2}^{-}+O \\ & {O}_{2}+{O}_{2}+e\to {O}_{2}+{O}_{2}^{-} \end{aligned}$$

**Figure 9:**
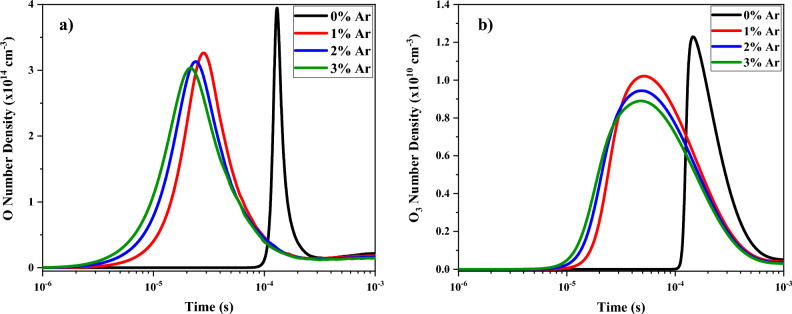
Temporal variations of density profile for (**a**) O and (**b**) O_3_ at various percentages of oxygen mixed by argon.

The values of O number density at different contents of argon added to oxygen plasma are represented in Fig. 9a. The O is produced mainly by the dissociation of the oxygen molecules by electron collision:

48 $$\begin{aligned} & e+{O}_{2}\to {O}^{-}+O \\ & e+{O}_{2}\to e+2O\left(P\right) \\ & e+{O}_{2}\to e+e+O+{O}^{+} \end{aligned}$$

and recombination of the O^−^ negative ions, due also to the electron collisions:

49 $$e+{O}^{-}\to e+e+O$$

So, it presents a maximum as the electron starts to increase and decrease similarly to the electron density. It can be seen that as soon as the oxygen atoms are created, their positive ions begin to produce in the plasma discharge by ionization and as they increase, the oxygen atoms are reduced:

50 $$e+O\to e+e+{O}^{+}$$

Again, this maximum moves to the lower times when the Ar concentration in the O_2_/Ar mixture is increased.

Figure 9b shows the temporal variations of number density of neutral reactive species, O and O_3_. It should be noted the high ozone production in this plasma discharge. This specie presents the highest density of all oxidative species formed with a maximum value near 10^10^ cm^−3^. This fact is very interesting for the possible application of this plasma discharge. As can be seen, the number density of O_3_ species has lower peak when the argon is added to the oxygen plasma, in respect to the pure oxygen. But, the durability of the ozone species is significantly increased at more contents of argon in the O_2_/Ar gas mixture. Also, the ozone species in the oxygen plasma mixed by argon gas is produced very earlier than the pure one. The ozone species is produced by the atomic collisions of oxygen atoms and ions with molecules:

51 $$\begin{aligned} & {O}^{-}+{O}_{2}\to {O}_{3}+e \\ & {O}^{-}+{O}_{2}^{a}\to {O}_{3}+e \\ & {O}_{2}^{-}+O\to e+{O}_{3} \end{aligned}$$

and destroyed mainly by the electron dissociation:

52 $$e+{O}_{3}\to {O}_{2}^{-}+O$$

### Spatial distribution of species in the plasma

The study of the spatial distribution of plasma parameter and species allow us to know where the species are formatted and to understand why this reactor design is suitable for the production of reactive oxides species. A 2-D distribution of species and plasma parameters are calculated for times 1 µs, where the plasma starts its formation and 1 ms, and we can consider that is it stabilized.

**Figure 10:**
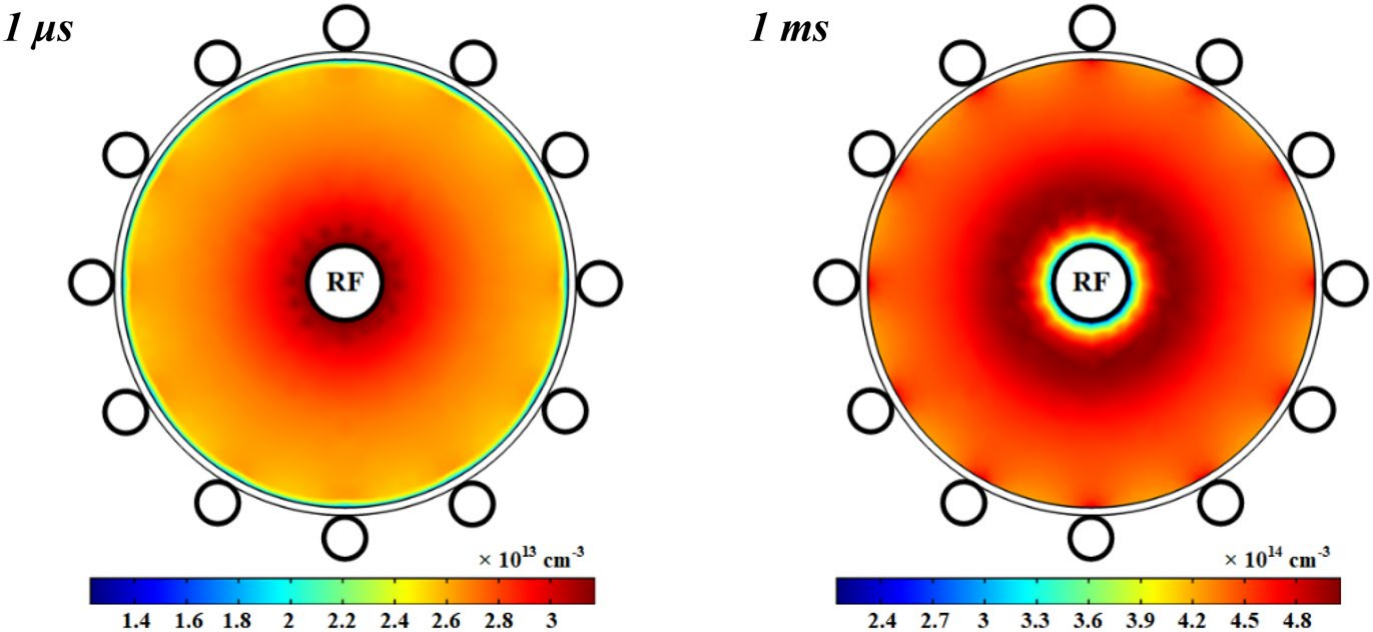
Spatial variations of density profiles for electrons at 1 μs and 1 ms with pure oxygen.

Figure 10 shows the variations of electrons density in the wire-to-multiwire DBD plasma with pure oxygen after 1 μs and 1 ms. The number density of electrons has a sharp peak close to the power electrode at the time of 1 μs and then, the number density drops in this region and the electrons travel along the radius to the center of tube as observed in the time of 1 ms. This is due to the fact that the electron collision ionization and dissociative ionization impacts are the main processes that create the electrons in this DBD plasma. Moreover, the cross-section of the collision and dissociative of electrons is more related to the electron temperature. The profile of electron temperature has a maximum value close to the ground electrode covered by dielectric in respect to other points near to dielectric (see Fig. 12), so the number density of electrons is higher in this region than other points on the dielectric. It should be mentioned that the electrons are easily diffused in the whole tube volume due to their lighter weight in respect to other species with the diffusion coefficient ${D}_{e}={k}_{B}{T}_{e}/{m}_{e}{v}_{c}$, where *k*_*B*_ is the Boltzmann constant, *T*_*e*_ is the electron temperature, *m*_*e*_ is the electron mass, and *v*_*e*_ is the momentum transfer collision frequency (see Fig. 13).

**Figure 11:**
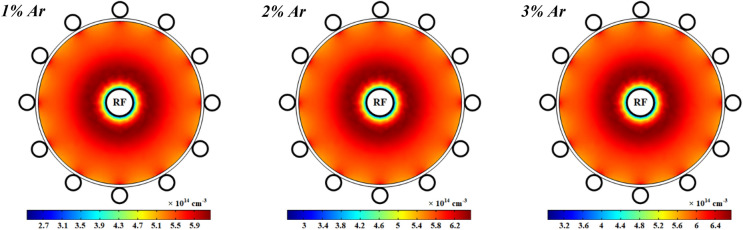
Spatial variations of density profiles for electrons at different percentage of argon and 1 ms.

Figures 11 represents the spatial variations of the electron number density in the plasma discharge medium at different fractions of argon added to the oxygen plasma. It is shown that the electrons number density is increased at more contributions of argon in the O_2_/Ar gas mixture and it reaches to a maximum in a ring shape near the power electrode while it drops at the closet to the power electrode. The argon atoms in the meta-stable state, Ar^*^, is one of the important species to additionally produce electrons in this case of plasma that is quenched by O_2_, and hence the electrons number density is increased by more adding Ar to the O_2_ plasma. On the other hand, the attachment process and dissociative attachment reactions with neutral radicals decrease the electrons in the plasma with pure oxygen gas and smaller contents of Ar in the O_2_/Ar gas mixture.

**Figure 12:**
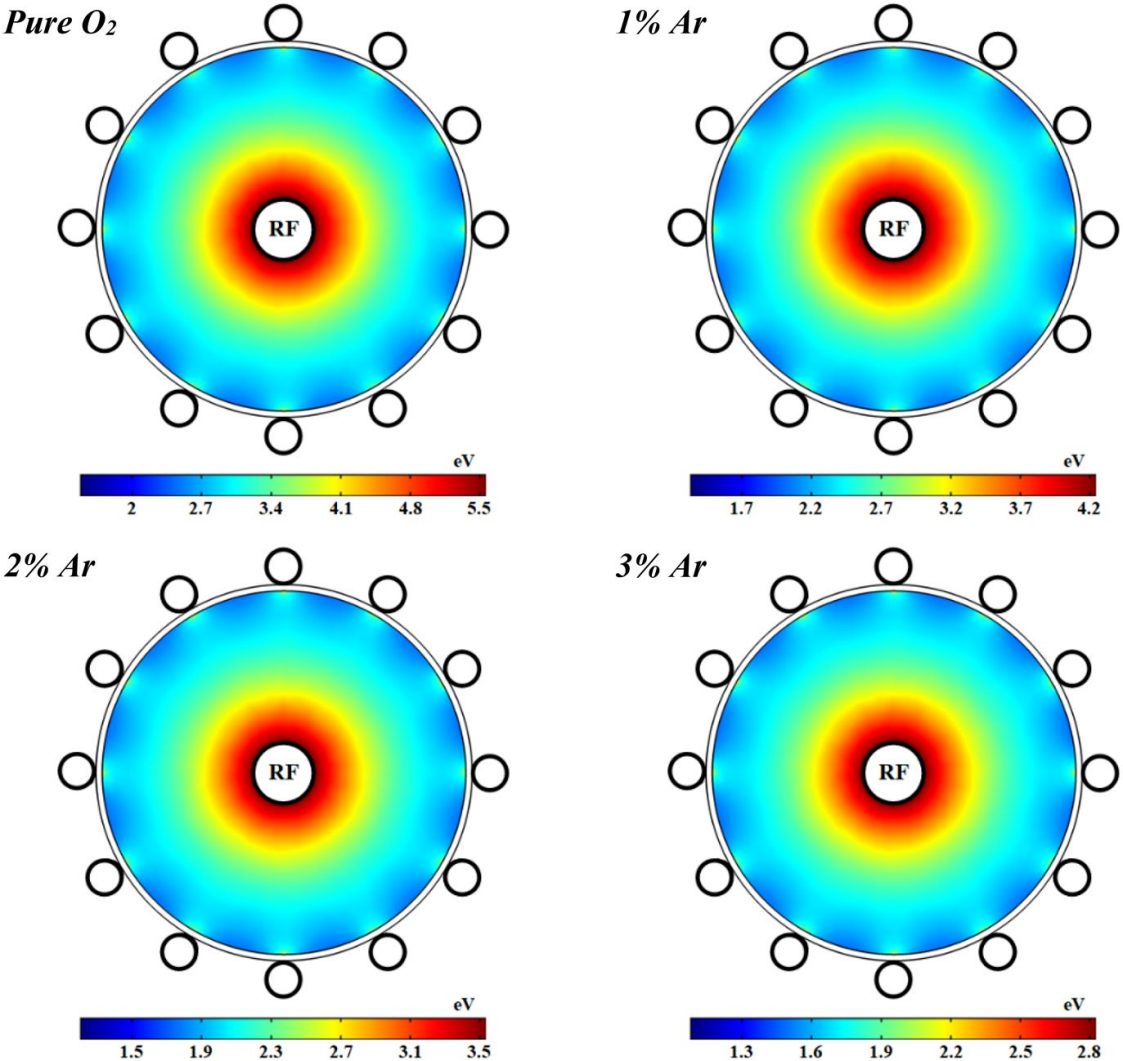
Spatial variations of temperature profiles for electrons at different percentage of argon and 1 ms.

Moreover, the profile of electrons temperature is shown in Fig. 12 at 1 kW RF power with pure oxygen and various percentages of argon mixed by oxygen. As can be seen, the electrons temperature is notably decreased by adding argon to the oxygen plasma. The electrons temperature has a peak close to the power electrode and it gradually drops along the radius of tube except close to the ground electrodes which is higher than other points on the dielectric. The electric field induced by RF power is stronger close to the power electrode (see Fig. 18), and thus the electrons get more energy there, and this results in the more electron temperature in this region. However, the electron temperature is decreased in the regions which the electron collision frequency is higher and therefore the diffusion is lower, because of the lack of electron heating. Besides, the loss of collision energy per electron–ion pair reduces with adding and increasing the argon gas to the oxygen plasma discharge. Furthermore, the electrons will receive the additional energy loss at smaller argon contributions in the O_2_/Ar gas mixture, and so the electron temperature grows up.

**Figure 13:**
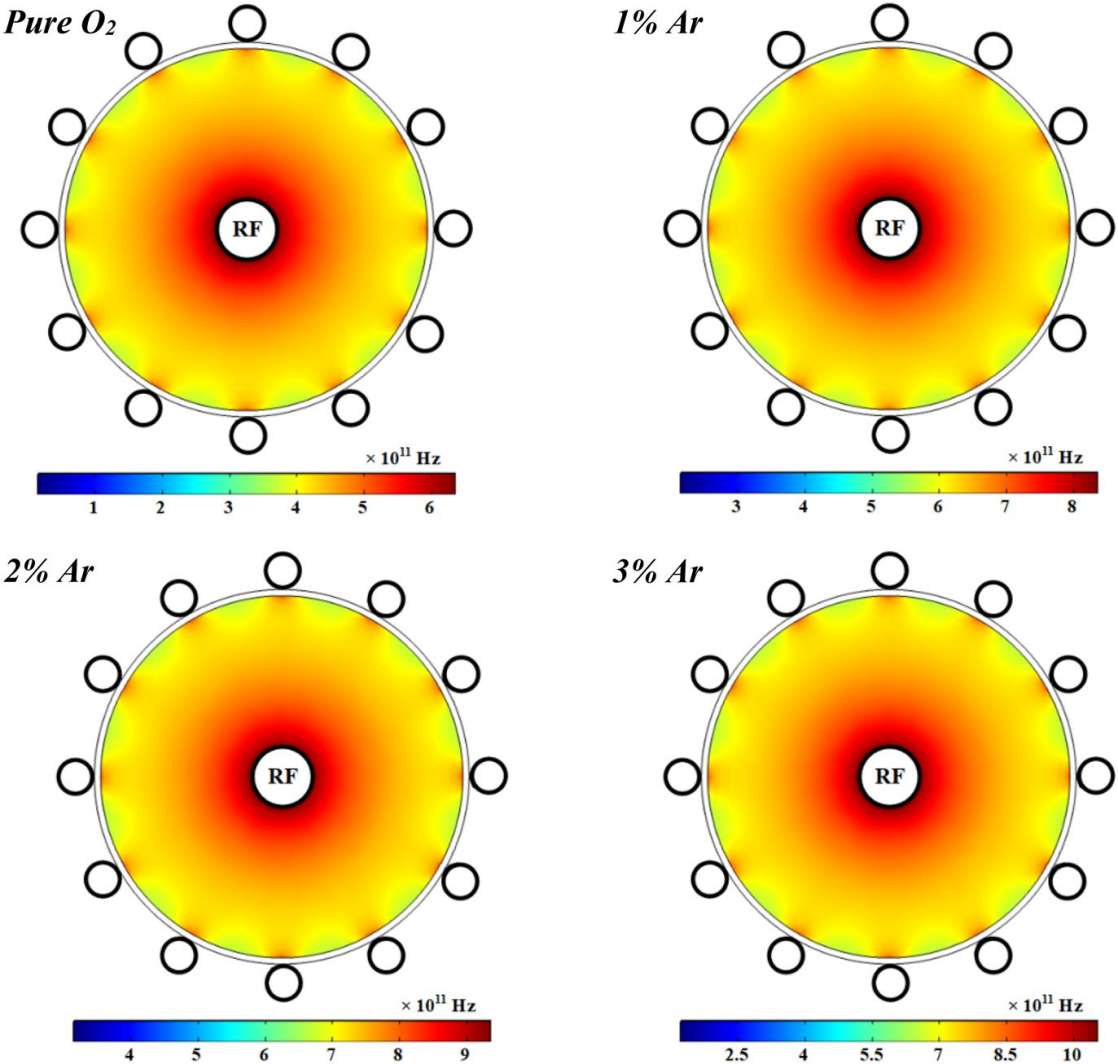
Spatial variations of plasma (electron) frequency profiles for electrons at different percentage of argon at 1 ms.

Figure 13 illustrates the collision frequency of electrons in the wire-to-multiwire dielectric barrier discharge at the RF power of 1 kW with pure oxygen and different contents of argon gas added to the oxygen plasma. As seen, the electron collision frequency is increased by adding argon gas to the oxygen plasma due to increase of electron number density in the plasma medium. The profile of collision frequency of electrons has a sharp peak near the central electrode and other peaks close to the ground electrodes. The electrons in these regions have less diffusivity in respect to other points in the plasma medium.

**Figure 14:**
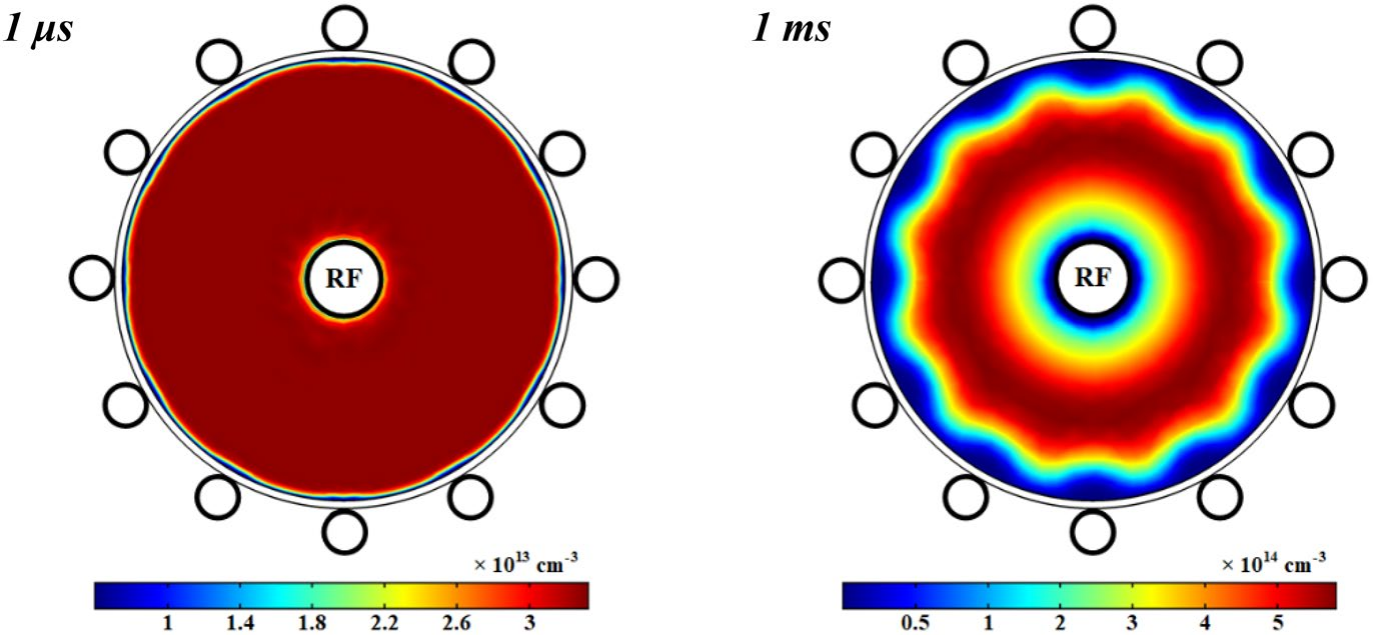
Spatial variations of density profile for positive ions at 1 μs and 1 ms with pure oxygen.

The changes of the number density of positive ions in the wire-to-multiwire DBD plasma with pure oxygen at 1 kW RF power after the time of 1 μs and 1 ms are presented in Fig. 14. It clear that the positive ions have radially moved in the plasma from both dielectric and power electrode to the center of tube at the time of 1 ms although they were almost diffused in all the plasma region at 1 μs.

**Figure 15:**
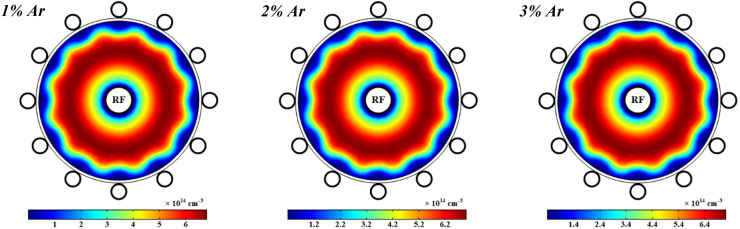
Spatial variations of density profile for positive ions at different percentage of argon after 1 ms.

Moreover, the spatial variations of the number density of positive ions in the considered plasma discharge at different contents of argon in the gas mixture with the RF power of 1 kW are shown in Fig. 15. It can be observed that the number density of positive ions increases at the higher argon percentages in O_2_/Ar gas mixture. This is owing to the higher cross section of momentum transfer for the atomic argon^56,57^, since collision energy of electrons decreases at the higher argon percentages. So, the recombination process between the electrons and positive ions will slightly occur in the plasma. It must be noted that, in addition to presence of ${O}^{+}$ and ${O}_{2}^{+}$, one of the most important positive ions is Ar^+^ in this case of plasma (see Table S1) which its density will be increased by adding argon gas to the oxygen plasma and rising its contents.

**Figure 16:**
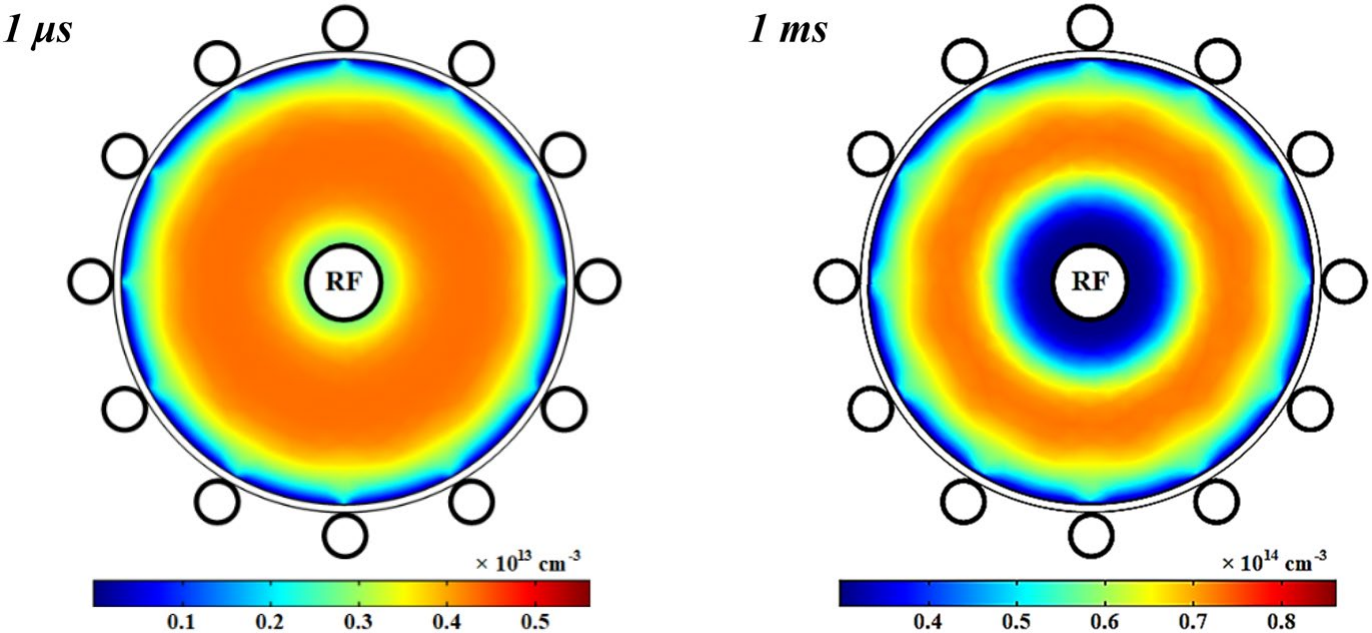
Spatial variations of density profile for negative ions at 1 μs and 1 ms with pure oxygen.

In addition, the negative ions also have the same behavior as the positive ions (see Fig. 13) near the power electrode in spite of the fact that they show an opposite behavior in respect to the dielectric wall as seen in Fig. 16. As can be seen the negative ions are also tending toward the ground electrode similar to the electrons (see Fig. 10) while the positive ions are depleting around the ground electrode. All the charged species have depleted around the power electrode after 1 ms. In addition, the number density of negative ions is lower than the electron number density (see Fig. 10), both of them are lower than positive ions due to quasi-neutrality condition.

As presented in Fig. 16, the negative ions in the plasma are highly confined in the bulk region with a circular shape. The trapping effect of positive plasma potential is able to create this confinement of negative ions in the case of plasma. It should be mentioned that the electron attachment is mainly responsible for producing the negative species (see Table S1), and therefore the negative ions distribution has a similar behavior by the spatial distribution of electrons density in the considered plasma. The electronegativity of negative ions is equal to 0.14 by considering the maximum values of electrons (4.9 × 10^14^ cm^−3^) and negative ions (0.7 × 10^14^ cm^−3^) in pure oxygen plasma at 1 ms if the relation of $\alpha ={n}_{-}/{n}_{e}$ is used.

**Figure 17:**
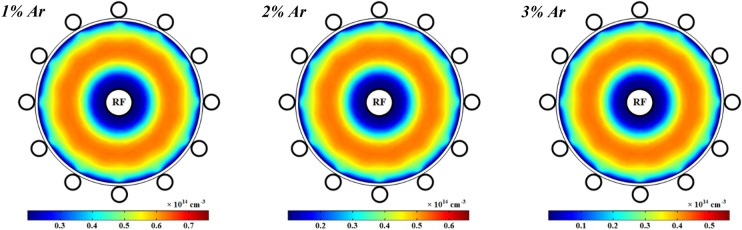
Spatial variations of density profile for negative ions at different percentage of argon after 1 ms.

Meanwhile, the spatial changes of the number density of negative ions in the wire-to-multiwire DBD plasma at various contributions of argon in the O_2_/Ar gas mixture and 1 kW RF power is introduced in Fig. 17. It is obvious that the number density of negative ions decreases at the more argon contents in O_2_/Ar gas mixture. It must be noted that, due to the electronegativity of oxygen, the negative ions are created by the dissociative attachment and electron impact detachment reactions. However, this is an important loss mechanism for negative ions in all the oxygen plasma discharges. Although the negative ions density is affected by the plasma electrons density due to the dissociative attachment reactions, the amount of oxygen in the plasma discharge medium is reduced by increasing argon gas. Therefore, the number density of negative ions decreases at the more Ar contents in the O_2_/Ar gas mixture.

Despite of the fact that the negative ions and electrons move to the dielectric wall during the time, the positive ions are accumulated at the radial center of tube and go away from the power electrode and dielectric wall. Moreover, the spatial distributions of all species become slightly asymmetrical during the time as observed in Figs. 10, 14, and 16. This owing to the fact that the electron heating will be more local during the time which results in the noticeable ionization near to the dielectric layer, and this causes the slightly asymmetrical distribution. Furthermore, the diffusion is reduced in this case of plasma because of working at atmospheric pressure. On the other hand, to reach a uniform and center-peaked distribution of charged species, the electron diffusion process and the loss of electrons in the attachment mechanism should be balanced in the oxygenate plasma that is not established in the wire-to-multiwire DBD plasma.

**Figure 18:**
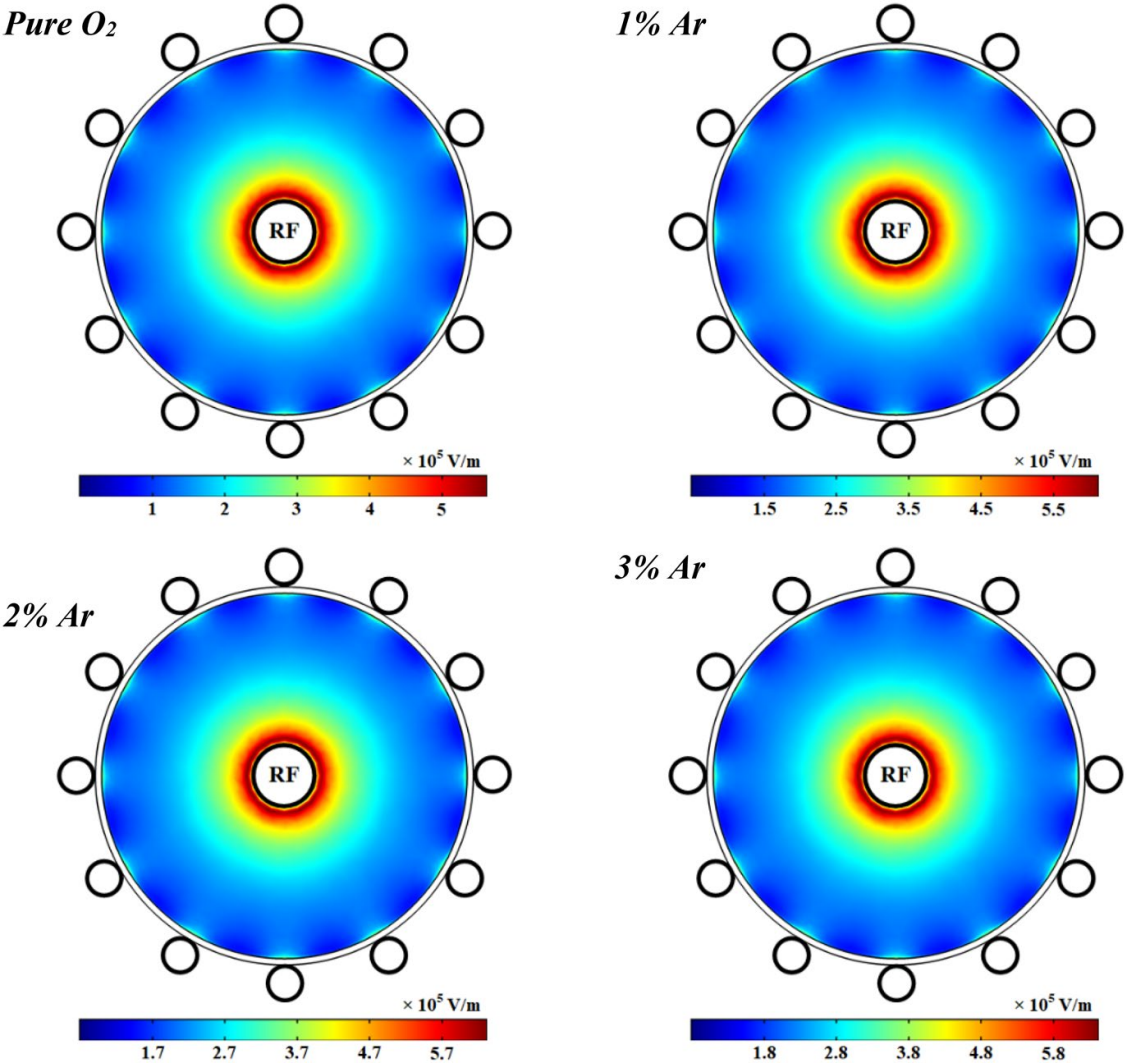
Spatial variations of electric field profile at pure oxygen and different percentage of argon after 1 ms.

The profile of the electric field in the wire-to-multiwire dielectric barrier discharge at 1 kW RF power with pure oxygen and different argon added to the oxygen plasma is illustrated in Fig. 18. It can be seen that the electric field has a maximum value close to the power electrode and it gradually decreases with the distance from the power electrode. Additionally, the electric field is slightly increased at higher percentages of argon gas in the mixture due to the increase of charged particles in the plasma medium.

## Conclusions

In this work, a new plasma jet was introduced by a wire-to-multiwire configuration and its operation in RF regime was numerically studied. Since it can assume that a wire consists of a large number of aligned points in a line, a wire is more singular than a surface. Therefore, the geometry used in designing this jet, i.e., wire-to-multiwire, is able to produce stronger electric field in relative to the wire-to-cylinder structure. It was supposed that this configuration operates by pure oxygen gas and its admixture with small amounts of argon at atmospheric pressure after applying a 1 kW RF power with frequency of 13.56 MHz. From the practical point of view, a dielectric layer of quartz was used to prevent the creation of shockwave in the structure and to obtain a stabilize plasma. Therefore, the plasma was studied in the wire-to-multiwire DBD configuration.

In addition, different species of oxygen and argon generated in the plasma jet was identified by OES technique. Due to reveal the ·OH radicals at the wavelength of 309.1 nm (${{\text{A}}}^{2}\Sigma \to {{\text{X}}}^{2}\Pi$ transition) in the plasma discharge as impurity, this band was used to obtain the rotation, vibration, and excitation temperatures of the plasma. On the other hand, the hydrogen Balmer β-series at wavelength of 486.1 nm was applied to calculate the electron density. It was found that all temperatures were decreased by adding argon and increasing its contribution to the gas mixture while the electron density was grown.

The formation of species in the discharge was studied by a global model. Temporal results show that the electron density largely determines the behavior of the formed species in the plasma, because the reactions kinetic is govern by electron collisions. The influence of Ar concentration on the species behavior was also studied by this model. The density of electrons and positive ions, i.e., ${O}^{+}$ and ${O}_{2}^{+}$, have been increased by adding more argon to the oxygen plasma while the density of negative ions, i.e., ${O}^{-}$ and ${O}_{2}^{-}$, and reactive neutral species, i.e., O and O_3_, have been decreased. This reduction is not significant and due to the decrease of oxygen content in the plasma medium. However, it was found that the durability of all oxygen species, i.e., reactive neutral species, positive and negative ions, was remarkably increased by adding argon to the oxygen plasma. The increase of durability is the most important result in many application areas that need to keep more oxidative species, especially in the medical applications.

This structure was also simulated through a 2D fluid scheme based on drift–diffusion model. The equations and boundary conditions required to simulate the introduced configuration were presented in detail. As regard the spatially results, the density of electrons and positive ions was significantly raised at presence of argon in the oxygen plasma while the negative ions density was reduced. This reduction was due to the decrease of oxygen amount in the plasma discharge medium that able to create the negative ion. Moreover, the density profiles of electron and negative ions showed that negative species are highly distanced from the power electrode after the time of 1 ms and accumulate near the center of the plasma tube and the ground electrodes. On the other hand, the positive species moved to the center of plasma tube from the central electrode and dielectric wall duration the time. The electron temperature profile showed that the electron heating will be more local during the time which result in the noticeable ionization near to the dielectric layer, and this causes the slightly asymmetrical distribution. Furthermore, the profile of the electron collision frequency showed that the diffusion is reduced in this case of plasma because of operating at atmospheric pressure. However, the equilibrium between the electron diffusion process and the loss of electrons in the attachment mechanism must be established in the oxygenate plasma to reach a uniform and center-peaked distribution of charged species that is not satisfied in the wire-to-multiwire DBD plasma. The simulation results of electron density and temperature in the wire-to-multiwire DBD plasma were compared by the experimental ones that was observed a good agreement between them.

### Supplementary Information

Supplementary Table S1.

## Data Availability

The data that support the findings of this study are available from the corresponding author upon reasonable request.

## References

[1] Murakami T, Kuroda SI, Osawa Z (1998). Dynamics of polymeric solid surfaces treated with oxygen plasma: Effect of aging media after plasma treatment. J. Colloid Interface Sci..

[2] Kim MC, Yang SH, Boo JH, Han JG (2003). Surface treatment of metals using an atmospheric pressure plasma jet and their surface characteristics. Surf. Coat. Technol..

[3] Strobel M, Lyons CS, Strobel JM, Kapaun RS (1992). Analysis of air-corona-treated polypropylene and poly (ethylene terephthalate) films by contact-angle measurements and X-ray photoelectron spectroscopy. J. Adhes. Sci. Technol..

[4] Suzuki M, Kishida A, Iwata H, Ikada Y (1986). Graft copolymerization of acrylamide onto a polyethylene surface pretreated with glow discharge. Macromolecules..

[5] Mrsic I, Baeuerle T, Ulitzsch S, Lorenz G, Rebner K, Kandelbauer A, Chasse T (2021). Oxygen plasma surface treatment of polymer films: Pellethane 55DE and EPR-g-VTMS. Appl. Surf. Sci..

[6] Habib SB, Gonzalez E, Hicks RF (2010). Atmospheric oxygen plasma activation of silicon (100) surfaces. J. Vacuum Sci. Technol. A..

[7] Deng X, Shi J, Kong MG (2006). Physical mechanisms of inactivation of *Bacillus subtilis* spores using cold atmospheric plasmas. IEEE Trans. Plasma Sci..

[8] Jin Y, Ren C, Yang L, Zhang J, Wang D (2013). Atmospheric pressure plasma jet in Ar and O_2_/Ar mixtures: Properties and high performance for surface cleaning. Plasma Sci. Technol..

[9] Poll HU, Schladitz U, Schreiter S (2001). Penetration of plasma effects into textile structures. Surf. Coat. Technol..

[10] Shenton MJ, Stevens GC (2001). Surface modification of polymer surfaces: Atmospheric plasma versus vacuum plasma treatments. J. Phys. D..

[11] Huang WT, Li SZ (2009). Preliminary study on applications of an atmospheric-pressure argon plasma discharge with a single-electrode configuration. IEEE Trans. Plasma Sci..

[12] Abramzon N, Joaquin JC, Bray J, Brelles-Mariño G (2006). Biofilm destruction by RF high-pressure cold plasma jet. IEEE Trans. Plasma Sci..

[13] Laroussi M, Hynes W, Akan T, Lu X, Tendero C (2008). The plasma pencil: A source of hypersonic cold plasma bullets for biomedical applications. IEEE Trans. Plasma Sci..

[14] Shao T, Zhang C, Long K, Zhang D, Wang J, Yan P, Zhou Y (2010). Surface modification of polyimide films using unipolar nanosecond-pulse DBD in atmospheric air. Appl. Surf. Sci..

[15] Shao T, Yu Y, Zhang C, Jiang H, Yan P, Zhou Y (2011). Generation of atmospheric pressure plasma by repetitive nanosecond pulses in air using water electrodes. Plasma Sci. Technol..

[16] Shao T, Zhang C, Niu Z, Yu Y, Yan P, Zhou Y (2011). Nanosecond repetitively pulsed dielectric barrier discharge in air at atmospheric pressure. Plasma Sci. Technol..

[17] Takechi K, Lieberman MA (2001). Effect of Ar addition to an O_2_ plasma in an inductively coupled, traveling wave driven, large area plasma source: O_2_/Ar mixture plasma modeling and photoresist etching. J. Appl. Phys..

[18] Tomanková K, Kubečka M, Rivolta N, Cornil D, Obrusník A (2023). Simulation of a hollow-cathode PECVD process in O_2_/TMDSO for silicon dioxide deposition: Cross-code validation of 2D plasma model and global plasma model. Surf. Coat. Technol..

[19] Brandenburg R (2017). Dielectric barrier discharges: Progress on plasma sources and on the understanding of regimes and single filaments. Plasma Sources Sci. Technol..

[20] Moravej M, Yang X, Hicks RF, Penelon J, Babayan SE (2006). A radio-frequency nonequilibrium atmospheric pressure plasma operating with argon and oxygen. J. Appl. Phys..

[21] Hsu CC, Nierode MA, Coburn JW, Graves DB (2006). Comparison of model and experiment for Ar, Ar/O_2_ and Ar/O_2_/Cl_2_ inductively coupled plasmas. J. Phys. D..

[22] Moss MS, Yanallah K, Allen RW, Pontiga F (2017). An investigation of CO2 splitting using nanosecond pulsed corona discharge: Effect of argon addition on CO_2_ conversion and energy efficiency. Plasma Sources Sci. Technol..

[23] Brezmes ÁO, Breitkopf C (2019). Numerical analysis of atmospheric pressure plasma produced by a dielectric barrier discharge in a mixture of Ar/CO_2_. IEEE Trans. Radiat. Plasma Med. Sci..

[24] Dorai R (2002). Modeling of Atmospheric Pressure Plasma Processing of Gases and Surfaces.

[25] Shrestha R, Subedi DP, Gurung JP, Wong CS (2016). Generation, characterization and application of atmospheric pressure plasma jet. Sains Malay..

[26] Raju GG (2006). Gaseous Electronics Theory and Practice.

[27] Laux CO, Spence TG, Kruger CH, Zare RN (2003). Optical diagnostics of atmospheric pressure air plasmas. Plasma Sources Sci. Technol..

[28] Chantry PJ (1987). A simple formula for diffusion calculations involving wall reflection and low density. J. Appl. Phys..

[29] Lieberman MA, Lichtenberg AJ (2005). Principles of plasma discharges and materials processing. MRS Bull..

[30] Godyak VA (1986). Soviet Radio Frequency Discharge Research.

[31] Hagelaar GJ, Pitchford LC (2005). Solving the Boltzmann equation to obtain electron transport coefficients and rate coefficients for fluid models. Plasma Sources Sci. Technol..

[32] Graef, W. A. D. *Zero-Dimensional Models for Plasma Chemistry*. Ph.D. thesis Eindhoven University of Technology (2012).

[33] Lieberman MA, Gottscho RA (1994). Physics of Thin Films.

[34] Chiper AS, Chen W, Mejlholm O, Dalgaard P, Stamate E (2011). Atmospheric pressure plasma produced inside a closed package by a dielectric barrier discharge in Ar/CO_2_ for bacterial inactivation of biological samples. Plasma Sources Sci. Technol..

[35] Raizer YP, Kisin VI, Allen JE (1991). Gas Discharge Physics.

[36] Popov NA (2016). Pulsed nanosecond discharge in air at high specific deposited energy: Fast gas heating and active particle production. Plasma Sources Sci. Technol..

[37] Popov NA (2011). Fast gas heating in a nitrogen–oxygen discharge plasma: I. Kinetic mechanism. J. Phys. D..

[38] Zhu Y, Wu Y, Cui W, Li Y, Jia M (2013). Numerical investigation of energy transfer for fast gas heating in an atmospheric nanosecond-pulsed DBD under different negative slopes. J. Phys. D..

[39] Becker MM, Loffhagen D, Schmidt W (2009). A stabilized finite element method for modeling of gas discharges. Comput. Phys. Commun..

[40] Hagelaar GJ, De Hoog FJ, Kroesen GM (2000). Boundary conditions in fluid models of gas discharges. Phys. Rev. E..

[41] Barkhordari A, Karimian S, Rodero A, Krawczyk DA, Mirzaei SI, Falahat A (2021). Carbon dioxide decomposition by a parallel-plate plasma reactor: Experiments and 2-D modelling. Appl. Sci..

[42] Nikiforov AY, Leys C, Gonzalez MA, Walsh JL (2015). Electron density measurement in atmospheric pressure plasma jets: Stark broadening of hydrogenated and non-hydrogenated lines. Plasma Sources Sci. Technol..

[43] Machala Z, Janda M, Hensel K, Jedlovský I, Leštinská L, Foltin V, Martišovitš V, Morvova M (2007). Emission spectroscopy of atmospheric pressure plasmas for bio-medical and environmental applications. J. Mol. Spectrosc..

[44] Fridman A (2008). Plasma Chemistry.

[45] Griem HR (1964). Plasma Spectroscopy.

[46] Boffard JB, Lin CC, DeJoseph CA (2004). Application of excitation cross sections to optical plasma diagnostics. J. Phys. D..

[47] Pandhija S, Rai AK (2009). In situ multielemental monitoring in coral skeleton by CF-LIBS. Appl. Phys. B..

[48] Belostotskiy SG, Ouk T, Donnelly VM, Economou DJ, Sadeghi N (2010). Gas temperature and electron density profiles in an argon dc microdischarge measured by optical emission spectroscopy. J. Appl. Phys..

[49] Lock EH, Fernsler RF, Slinker S, Walton SG (2011). Experimental and Theoretical Estimation of Excited Species Generation in Pulsed Electron Beam-Generated Plasmas Produced in Pure Argon, Nitrogen, Oxygen, and Their Mixtures.

[50] Straub HC, Renault P, Lindsay BG, Smith KA, Stebbings RF (1995). Absolute partial and total cross sections for electron-impact ionization of argon from threshold to 1000 eV. Phys. Rev. A..

[51] Belostotsky SG, Economou DJ, Lopaev DV, Rakhimova TV (2005). Negative ion destruction by O (3P) atoms and O2 (a 1Δg) molecules in an oxygen plasma. Plasma Sources Sci. Technol..

[52] Barkhordari A, Mirzaei SI, Falahat A, Rodero A (2021). Numerical and experimental study of an Ar/CO_2_ plasma in a point-to-plane reactor at atmospheric pressure. Spectrochim. Acta B..

[53] Shimamori H, Fessenden RW (1981). Thermal electron attachment to oxygen and van der Waals molecules containing oxygen. J. Chem. Phys..

[54] Midey AJ, Viggiano AA (1998). Rate constants for the reaction of Ar+ with O_2_ and CO as a function of temperature from 300 to 1400 K: Derivation of rotational and vibrational energy effects. J. Chem. Phys..

[55] Gaucherel P, Rowe B (1977). Measurement of rates of charge exchange and dissociative recombination reactions in Ar-N_2_, Ar-H_2_ and Ar-O_2_ mixtures. Int. J. Mass Spectrom. Ion Phys..

[56] Barkhordari A, Ganjovi A, Mirzaei I, Falahat A (2018). Study of the physical discharge properties of a Ar/O_2_ DC plasma jet. Indian J. Phys..

[57] Nikolić M, Sepulveda I, Gonzalez C, Khogeer N, Fernandez-Monteith M (2021). Applicability of optical emission spectroscopy techniques for characterization of Ar and Ar/O_2_ discharges. J. Phys. D..

