# Supplementary material for: Influence of the argon admixture on the reactive oxide species formation inside an atmospheric pressure oxygen plasma jet
Source: Sci Rep. 2024 Feb 10;14:3425. doi: 10.1038/s41598-024-54111-y (PMC10858918; doi:10.1038/s41598-024-54111-y)
Supplement: Supplementary file 1 — Supplementary Table S1. [file 41598_2024_54111_MOESM1_ESM.docx]

Online supporting information for the following article

**Influence of the Argon Admixture on the Reactive Oxide Species Formation inside an Atmospheric Pressure Oxygen Plasma Jet**

**Ali Barkhordari^1*^, Saeed Karimian^2^, Sajedeh Shahsavari^3^, Dorota Krawczyk^4^, Antonio Rodero^5^**

^1^ Faculty of Physics, Shahid Bahonar University of Kerman, Kerman, Iran

^2^ Department of Physics, Vali-e-Asr University of Rafsanjan, Rafsanjan, Iran

^3^ Photonics institute, Kerman Graduate University of Technology, Kerman, Iran

^4^ Faculty of Civil Engineering and Environmental Sciences, Bialystok University of Technology, Bialystok, Poland

^5^ Department of Physics, School of Engineering Sciences of Belmez, University of Cordoba, Cordoba, Spain

***Email:*** [alibarkhordari20@yahoo.com](mailto:alibarkhordari20@yahoo.com)

**Table S1.** List of the reactions considered in the model.

| **Num** | **Reaction** | **Rate Coefficient (m^3^.s^-1^ or m^6^.s^-1^)** | **Refs.** |
| --- | --- | --- | --- |
| 1 | $e+Ar\to e+Ar$ | $k_{1}$ | [50] |
| 2 | $e+Ar\to e+{Ar}^{*}$ | $k_{2}$ | [50] |
| 3 | $e+{Ar}^{*}\to e+e+{Ar}^{+}$ | $k_{3}$ | [50] |
| 4 | $e+{Ar}^{*}\to e+Ar$ | $k_{4}$ | [50] |
| 5 | $e+Ar\to e+e+{Ar}^{+}$ | $k_{5}$ | [50] |
| 6 | $e+O_{2}\to e+O_{2}$ | $4.7\times{10}^{-8}T_{e}^{0.5}$ | [50] |
| 7 | $e+O_{2}\to e+O_{2}^{a}$ | $1.7\times{10}^{-9}exp(-{3.1}/{T_{e}})$ | [50] |
| 8 | $e+O_{2}\to e+O^{-}+O^{+}$ | $7.1\times{10}^{-11}T_{e}^{0.5}exp(-{17}/{T_{e}})$ | [50] |
| 9 | $e+O_{2}\to O^{-}+O$ | $8.8\times{10}^{-11}exp(-{4.4}/{T_{e}})$ | [50] |
| 10 | $e+O_{2}\to e+2O\left( P \right)$ | $4.2\times{10}^{-9}T_{e}^{0.5}exp(-{5.6}/{T_{e}})$ | [50] |
| 11 | $e+O_{2}\to e+e+O+O^{+}$ | $5.3\times{10}^{-10}T_{e}^{0.9}exp(-{20}/{T_{e}})$ | [50] |
| 12 | $e+O\to e+e+O^{+}$ | $9\times{10}^{-9}T_{e}^{0.7}exp(-{13}/{T_{e}})$ | [51] |
| 13 | $e+O_{2}\to e+e+O_{2}^{+}$ | $9\times{10}^{-10}T_{e}^{0.5}exp(-{12.6}/{T_{e}})$ | [51] |
| 14 | $e+O_{2}\to e+O+O(D)$ | $5\times{10}^{-8}exp(-{8.4}/{T_{e}})$ | [51] |
| 15 | $e+O_{2}\to e+2O$ | $4.2\times{10}^{-15}exp(-{5.6}/{T_{e}})$ | [51] |
| 16 | $e+O+O_{2}\to O+O_{2}^{-}$ | $1.00\times{10}^{-43}$ | [51] |
| 17 | $e+O\to e+O(D)$ | $4.2\times{10}^{-9}exp(-{2.25}/{T_{e}})$ | [51] |
| 18 | $e+O(D)\to e+O$ | $8\times{10}^{-9}$ | [51] |
| 19 | $e+O(D)\to e+e+O^{+}$ | $9\times{10}^{-9}T_{e}^{0.7}exp(-{11.6}/{T_{e}})$ | [51] |
| 20 | $e+O_{2}^{+}\to O+O$ | $5.2\times{10}^{-9}/T_{e}$ | [51] |
| 21 | $e+O^{-}\to e+e+O$ | $2\times{10}^{-7}exp(-{5.5}/{T_{e}})$ | [52] |
| 22 | $e+O_{2}^{a}\to e+O_{2}$ | $5.6\times{10}^{-9}exp(-{2.2}/{T_{e}})$ | [52] |
| 23 | $e+O_{2}^{a}\to O+O$ | $2.28\times{10}^{-10}exp(-{2.29}/{T_{e}})$ | [52] |
| 24 | $e+O_{2}^{a}\to e+e+O_{2}^{+}$ | $9\times{10}^{-10}T_{e}^{2}exp(-{11.6}/{T_{e}})$ | [52] |
| 25 | $e+O_{2}^{a}\to e+2O$ | $4.2\times{10}^{-9}exp(-{4.6}/{T_{e}})$ | [52] |
| 26 | $e+O_{2}^{a}\to e+O+O(D)$ | $2.08\times{10}^{-8}exp(-{7.4}/{T_{e}})$ | [52] |
| 27 | $e+O_{2}^{a}\to e+e+O+O^{+}$ | $5.3\times{10}^{-10}T_{e}^{0.9}exp(-{19}/{T_{e}})$ | [52] |
| 28 | $O^{-}+O_{2}^{+}\to O_{2}+O$ | $1\times{10}^{-7}$ | [52] |
| 29 | $e+O_{3}\to O_{2}^{-}+O$ | $9.76\times{10}^{-14}T_{e}^{-1.309}exp(-{1.007}/{T_{e}})$ | [52] |
| 30 | $O^{-}+O_{2}^{+}\to3O$ | $1\times{10}^{-7}$ | [52] |
| 31 | $O^{-}+O^{+}\to2O$ | $2.7\times{10}^{-7}{({300}/{T_{g}})}^{0.5}$ | [52] |
| 32 | $O^{+}+O_{2}\to O_{2}^{+}+O$ | $2\times{10}^{-11}{({300}/{T_{g}})}^{0.5}$ | [52] |
| 33 | $O_{2}^{a}+O_{2}\to2O_{2}$ | $2.2\times{10}^{-18}{({300}/{T_{g}})}^{0.8}$ | [53] |
| 34 | $O_{2}^{a}+O\to O_{2}+O$ | $2\times{10}^{-16}$ | [53] |
| 35 | $O(D)+O\to2O$ | $8\times{10}^{-12}$ | [53] |
| 36 | $O(D)+O_{2}\to O+O_{2}$ | $7\times{10}^{-12}({67}/{T_{g}})$ | [53] |
| 37 | $O(D)+O_{2}\to O+O_{2}^{a}$ | $1\times{10}^{-12}$ | [53] |
| 38 | $O^{+}+O_{2}^{a}\to O+O_{2}^{+}$ | $2.1\times{10}^{-11}$ | [53] |
| 39 | $O^{-}+O_{2}^{a}\to e+O+O_{2}$ | $1\times{10}^{-10}{({300}/{T_{i}})}^{0.5}$ | [53] |
| 40 | ${Ar}^{+}+O_{2}\to Ar+O_{2}^{+}$ | $1.1\times{10}^{-10}$ | [54] |
| 41 | ${Ar}^{+}+O_{2}^{*}\to Ar+O_{2}^{+}$ | $1.1\times{10}^{-10}$ | [54] |
| 42 | ${Ar}^{+}+O\to Ar+O^{+}$ | $1.1\times{10}^{-10}$ | [54] |
| 43 | ${Ar}^{+}+O^{*}\to Ar+O^{+}$ | $1.1\times{10}^{-10}$ | [54] |
| 44 | ${Ar}^{+}+O^{-}\to Ar+O$ | $2.8\times{10}^{-7}$ | [54] |
| 45 | ${Ar}^{*}+O_{2}\to Ar+2O$ | $2.1\times{10}^{-16}$ | [54] |
| 46 | $Ar+O_{2}^{+}\to{Ar}^{+}+O_{2}$ | $5.5\times{10}^{-11}$ | [54] |
| 47 | $Ar+O_{3}\to O+O_{2}+Ar$ | $4.2\times{10}^{-16}exp(-{11430}/{T_{g}})$ | [54] |
| 48 | $e+O^{+}+O_{2}\to O+O_{2}$ | $6\times{10}^{-39}{({300}/{T_{e}})}^{1.5}$ | [54] |
| 49 | $Ar+O^{+}\to O+{Ar}^{+}$ | $3\times{10}^{-17}$ | [54] |
| 50 | $O^{+}+O_{3}\to O_{2}^{+}+O_{2}$ | $1\times{10}^{-16}$ | [55] |
| 51 | ${Ar}^{*}+Ar\to Ar+Ar$ | $2.3\times{10}^{-21}$ | [55] |
| 52 | ${Ar}^{*}+{Ar}^{*}\to Ar+{Ar}^{+}+e$ | $6.4\times{10}^{-16}$ | [55] |
| 53 | $O_{2}^{+}+O_{2}\to O_{2}^{+}+O_{2}$ | $3\times{10}^{-17}$ | [55] |
| 54 | $O^{-}+O_{2}\to O_{3}+e$ | $5\times{10}^{-21}$ | [55] |
| 55 | $O^{-}+O_{2}^{a}\to O_{3}+e$ | $3\times{10}^{-16}$ | [55] |
| 56 | ${Ar}^{+}+O_{2}^{a}\to Ar+O_{2}^{+}$ | $1.1\times{10}^{-16}$ | [55] |
| 57 | $O^{-}+O\to O_{2}+e$ | $5\times{10}^{-16}$ | [55] |
| 58 | ${Ar}^{*}+O_{2}\to Ar+O_{2}$ | $1.1\times{10}^{-9}$ | [55] |
| 59 | ${Ar}^{*}+O\to Ar+O$ | $8.1\times{10}^{-12}$ | [55] |
| 60 | $e+O_{3}\to O_{2}^{-}+O$ | $1\times{10}^{-9}$ | [55] |
| 61 | $O^{-}+O_{2}^{*}\to O_{2}^{-}+O$ | $1\times{10}^{-10}$ | [54] |
| 62 | $O_{2}+O_{2}+e\to O_{2}+O_{2}^{-}$ | $2\times{10}^{-41}({300}/{T_{e}})$ | [52] |
| 63 | $O_{2}^{-}+O^{+}\to O_{2}+O$ | $2\times{10}^{-13}({300}/{T_{g}})$ | [53] |
| 64 | $O_{2}^{-}+O_{2}^{+}\to O_{2}+O_{2}$ | $2\times{10}^{-13}$ | [53] |
| 65 | $O_{2}^{-}+O_{2}^{+}\to O+O+O_{2}$ | $4.2\times{10}^{-13}$ | [53] |
| 66 | $O_{2}^{-}+O_{2}^{+}+O_{2}\to O_{2}+O_{2}+O_{2}$ | $2\times{10}^{-25}$ | [53] |
| 67 | $O_{2}^{-}+O\to e+O_{3}$ | $5\times{10}^{-18}{({300}/{T_{g}})}^{-0.5}$ | [52] |
| 68 | $O^{-}+O_{3}\to O_{2}^{-}+O_{2}$ | $1.02\times{10}^{-17}{({300}/{T_{g}})}^{-0.5}$ | [54] |
| 69 | $O_{3}^{-}+O\to O_{2}^{-}+O_{2}$ | $2.5\times{10}^{-16}{({300}/{T_{g}})}^{-0.5}$ | [52] |
